# A Multi-Country Cross-Sectional Study of Vaginal Carriage of Group B Streptococci (GBS) and *Escherichia coli* in Resource-Poor Settings: Prevalences and Risk Factors

**DOI:** 10.1371/journal.pone.0148052

**Published:** 2016-01-26

**Authors:** Piet Cools, Vicky Jespers, Liselotte Hardy, Tania Crucitti, Sinead Delany-Moretlwe, Mary Mwaura, Gilles F. Ndayisaba, Janneke H. H. M. van de Wijgert, Mario Vaneechoutte

**Affiliations:** 1 Laboratory Bacteriology Research, Department of Microbiology, Immunology, and Clinical Chemistry, Faculty of Medicine and Health Sciences, Ghent University, Ghent, Belgium; 2 Unit of Epidemiology and Control of HIV/STD, Department of Public Health, Institute of Tropical Medicine, Antwerp, Belgium; 3 HIV/STI Reference Laboratory, Department of Clinical Sciences, Institute of Tropical Medicine, Antwerp, Belgium; 4 Wits Reproductive Health & HIV Institute, University of Witwatersrand, Johannesburg, South Africa; 5 ICRH Kenya, Mombasa, Kenya; 6 Rinda Ubuzima, Kigali, Rwanda; 7 Department of Clinical Infection, Microbiology and Immunology, Institute of Infection and Global Health, University of Liverpool, Liverpool, United Kingdom; University of Cambridge, UNITED KINGDOM

## Abstract

**Background:**

One million neonates die each year in low- and middle-income countries because of neonatal sepsis; group B *Streptococcus* (GBS) and *Escherichia coli* are the leading causes. In sub-Saharan Africa, epidemiological data on vaginal GBS and *E*. *coli* carriage, a prerequisite for GBS and *E*. *coli* neonatal sepsis, respectively, are scarce but necessary to design and implement prevention strategies. Therefore, we assessed vaginal GBS and *E*. *coli* carriage rates and risk factors and the GBS serotype distribution in three sub-Saharan countries.

**Methods:**

A total of 430 women from Kenya, Rwanda and South Africa were studied cross-sectionally. Vaginal carriage of GBS and *E*. *coli*, and GBS serotype were assessed using molecular techniques. Risk factors for carriage were identified using multivariable logistic regression analysis.

**Results:**

Vaginal carriage rates in reference groups from Kenya and South Africa were 20.2% (95% CI, 13.7–28.7%) and 23.1% (95% CI, 16.2–31.9%), respectively for GBS; and 25.0% (95% CI, 17.8–33.9%) and 27.1% (95% CI, 19.6–36.2%), respectively for *E*. *coli*. GBS serotypes Ia (36.8%), V (26.3%) and III (14.0%) were most prevalent. Factors independently associated with GBS and *E*. *coli* carriage were *Candida albicans*, an intermediate vaginal microbiome, bacterial vaginosis, recent vaginal intercourse, vaginal washing, cervical ectopy and working as a sex worker. GBS and *E*. *coli* carriage were positively associated.

**Conclusions:**

Reduced vaginal GBS carriage rates might be accomplished by advocating behavioral changes such as abstinence from sexual intercourse and by avoidance of vaginal washing during late pregnancy. It might be advisable to explore the inclusion of vaginal carriage of *C*. *albicans*, GBS, *E*. *coli* and of the presence of cervical ectopy in a risk- and/or screening-based administration of antibiotic prophylaxis. Current phase II GBS vaccines (a trivalent vaccine targeting serotypes Ia, Ib, and III, and a conjugate vaccine targeting serotype III) would not protect the majority of women against carriage in our study population.

## Introduction

One million children die each year in low- and middle-income countries in the first 4 weeks of life because of neonatal sepsis [1]. Early-onset neonatal sepsis (EOS), occurring in the first week of life, accounts for approximately 80% of cases, and is caused by bacteria that are transmitted vertically from the genital tract of the mother to infant before or during delivery [2]. Late-onset neonatal sepsis (LOS) occurs between week 1 and month 2 to 3 of life and may be caused by bacteria acquired vertically or horizontally [3]. Because the transfer of a single species from the maternal genitourinary tract to the neonate before or during delivery is a prerequisite for EOS [4], there are unique opportunities for prevention of EOS.

At present, *Streptococcus agalactiae* (Group B *Streptococcus*, GBS) and *Escherichia coli* are the leading causes of EOS worldwide [5]. Furthermore, GBS and *E*. *coli* are associated with preterm birth, very-low-birth-weight delivery and puerperal sepsis [6, 7], which cause substantial morbidity and mortality in sub-Saharan Africa (SSA) [2, 8, 9].

To prevent EOS, efforts have been focusing mainly on GBS and high-income countries, based on two strategies, namely the screening- or risk-based administration of intrapartum antibiotic prophylaxis (IAP) and the development of vaccines [10].

IAP has been shown to reduce the incidence of GBS EOS from 1.7/1000 to 0.6/1000 in the US [11], but is not effective against *E*. *coli* EOS, LOS, and adverse perinatal outcomes related to GBS [12, 13]. Furthermore, according to the current universal guidelines (Centers for Disease Control and Prevention, CDC), IAP should be administered to women found positive for GBS at 35–37 weeks of gestation [14]. However, these guidelines are not followed in most health-care facilities in low-income countries. The use of intravaginal washes with chlorhexidine (a wide-spectrum microbicide) during labour and neonatal wipes with chlorhexidine, has been explored in low- and middle-income countries, but is unlikely to prevent vertically acquired neonatal infections in any setting or population [4].

Most GBS vaccines under development aim at eliciting protective antibodies against capsular polysaccharides (CPS), the most important GBS virulence factor of which ten antigenically distinct CPS are known [10], and are attractive as some of the IAP-related problems may be circumvented [10]. However, these vaccines might not be effective in low-income countries because of different serotype distribution [15].

Although SSA has the highest rates of neonatal sepsis mortality worldwide, epidemiological data on vaginal GBS and *E*. *coli* carriage are very limited but necessary to develop and implement prevention strategies [16, 17]. Therefore, in this multi-country cross-sectional study, we assessed the vaginal GBS and *E*. *coli* carriage prevalence, risk factors for GBS and *E*. *coli* carriage, and GBS serotype distribution in populations from three countries: Kenya, Rwanda and South Africa.

## Patients and Methods

### Study design and population

In 2010–2011, we conducted a multi-country follow-up study entitled ‘‘Characterisation of novel microbicide safety biomarkers in East and South Africa”. The main aim of that project was to characterise the vaginal microbiome and the cervicovaginal mucosal immune system in African women and to assess changes of these over time [18–21]. In that study, 430 women were recruited at three study sites, i.e. the International Centre for Reproductive Health Kenya (ICRHK) in Mombasa, Kenya (170 women); the non-governmental organisation Rinda Ubuzima (RU) in Kigali, Rwanda (60 women), and the Wits Reproductive Health and HIV Institute (Wits RHI) in Johannesburg, South Africa (SA) (200 women). The women were recruited into 6 predefined study groups: a reference group of 219 women (adult, non-pregnant, HIV-negative women at average risk of HIV), 60 pregnant women (up to 14 weeks of gestational age as determined by abdominal ultrasound at recruitment), 60 adolescent girls (16–17 years), 31 HIV-negative women engaging in vaginal practices (usage of cloth, lemon juice, or detergents to clean, dry or tighten the vagina on a regular basis), 30 self-acknowledged female sex workers (FSW), and 30 HIV-positive women (on antiretroviral treatment for at least 6 months, asymptomatic and with a CD4 count of more than 350 cells/μl) (Table 1). Participants were eligible for inclusion if they were in good physical and mental health, able and willing to participate in the study as required by the protocol, able and willing to give written informed consent (including written parental or guardian consent for adolescents). Women were excluded if they had never had penetrative vaginal intercourse, if they had a history of hysterectomy or other genital tract surgery in the three months prior to the screening visit, if external and/or internal genital warts were found, if they were enrolled in HIV prevention trials involving investigational products, if they were less than 6 months post-partum at the time of enrolment, if they were HIV-positive (unless for inclusion in the HIV-positive women group), or if they were pregnant (unless for inclusion in the pregnant women group). The study population, followed up for approximately eight months per person over 8 visits, is described in detail by Jespers and coworkers [19].

**Table 1:** Study population and vaginal GBS and *E*. *coli* carriage rates.

City, Country	Group	n	GBS prevalence % (95% CI)	*E*. *coli* prevalence % (95% CI)
**Mombasa, Kenya**	Reference group	110	20.2 (13.7–28.7)	25.0 (17.8–33.9)
**Mombasa, Kenya**	Pregnant women	30	14.3 (5.7–31.5)	14.3 (5.7–31.5)
**Mombasa, Kenya**	Adolescents	30	3.6 (0.6–17.7)	28.6 (15.3–47.1)
**Kigali, Rwanda**	FSW	30	20.0 (9.5–37.3)	70.0 (52.1–83.3)
**Kigali, Rwanda**	HIV+ women	30	0.0 (0.0–11.4)	20.0 (9.5–37.3)
**Johannesburg, SA**	Reference group	109	23.2 (16.2–31.9)	27.1 (19.6–36.2)
**Johannesburg, SA**	Pregnant women	30	10.0 (3.5–25.6)	33.3 (19.2–51.2)
**Johannesburg, SA**	Adolescents	30	0.0 (0.0–11.4)	13.3 (5.3–29.7)
**Johannesburg, SA**	Vaginal practices	31	25.8 (13.7–43.2)	30.0 (16.7–47.9)

The current study presents one of the tertiary objectives of the above mentioned study, namely to document the vaginal carriage rates of the main pathogens associated with EOS (GBS and *E*. *coli*) and the risk factors for their carriage. These analyses are based on the screening visit and the first visit (scheduled soon after the last day (day 9 +/- 2 days) of the menstrual period) from the follow-up study.

### Study procedures

At the screening visit, blood, vaginal, endocervical and urine samples were taken for diagnostic testing of HIV, HSV-2, syphilis, *Neisseria gonorrheae* (NG), *Chlamydia trachomatis* (CT), *Trichomonas vaginalis* (TV), urinary tract infection (UTI), pregnancy, cervical dysplasia (by Pap smear), bacterial vaginosis (BV) (Amsel criteria), and vaginal candidiasis. Treatment was provided according to national guidelines, voluntary HIV counseling was offered, and condoms were provided free-of-charge.

At visit 1, two sterile Copan flocked^®^ vaginal swabs (Copan Diagnostics, Inc., Murrieta, CA), to be used for the molecular detection of GBS and *E*. *coli*, were brought into the vaginal vault by the study clinician, rotated against the vaginal wall at the midportion of the vault, gently dipped in the posterior fornix and carefully removed to prevent contamination with the microbiome of the vulva and introitus. The swab heads were collected into two 1.5 ml cryovials, labelled and immediately frozen at -80°C until shipment to the central laboratory at the Institute of Tropical Medicine (ITM, Antwerp, Belgium) using temperature-monitored dry shippers filled with liquid nitrogen. One Amies swab (Copan Diagnostics, Inc.) for culturing was taken in a likewise manner, placed in the Amies tube, and transported at 4°C in a temperature-monitored cooler to the local laboratory, where it was processed immediately. At both visits, women were interviewed during face-to-face interviews about their general and sexual health, vaginal habits and sociodemographic characteristics. A physical examination including speculum and bimanual pelvic examination was carried out by a clinician. At each visit, participants were compensated for their time and transportation.

### Diagnosis of genital infections

At the local laboratories, tests for HIV, HSV-2, syphilis, CT and NG were performed. For immediate detection of *Candida* cells and hyphae, TV, and clue cells, wet mount microscopy was used. For the purpose of this study, a commercially available TV InPouch^TM^ system (BioMed Diagnostics, White City, Oregon) was used. For this, a vaginal swab was inoculated according to the manufacturer’s instructions and InPouch cultures were monitored on a daily basis. InPouch bags with no growth at the end of five days were considered negative. BV diagnosed according to the Amsel criteria was used for immediate treatment. For research, vaginal smears were made and sent to ITM for Gram staining and Nugent scoring, a scoring system to diagnose BV. Briefly, smears from vaginal swabs were prepared by rolling the swab onto a glass slide. Slides were air-dried and fixed using 70% ethanol. For the Gram-staining at ITM, the fixed smear was covered with crystal violet for 1 minute, washed with water, flooded with Lugol’s iodine for 1 minute, washed with water, and then decolorized with acetone-alcohol for 2–3 seconds. The smears were rinsed quickly under running water to stop the decolorisation and then counterstained with safranin for 1 minute. All reagents were from Becton Dickinson (BD). All smears were examined microscopically with the 40X objective to check the staining and the distribution of the material, and then assessed under oil immersion objective (1000x magnification) using the grading system described by Nugent and co-workers [22]. The Nugent score is calculated by assessing for the presence of *Lactobacillus* cell types, small Gram-variable coccobacilli, and curved Gram-variable rods. A score of 0–3 is considered as normal (BV-negative); a score of 4–6 as an intermediate vaginal microbiome; and a score of 7–10 as BV-positive.

### DNA extraction

For the molecular detection of GBS and *E*. *coli*, DNA extraction from the two Copan swabs of each subject was carried out at ITM by thawing the swabs at room temperature for 30 minutes. After adding 1200 μL of diluted PBS, each swab was gently vortexed for 15 seconds, and 1 mL of each swab suspension was pooled into a final volume of 2 mL. An aliquot of 250 μL was extracted using the Abbott m24sp automated extraction platform (Abbott, Maidenhead, UK), according to the manufacturer’s instructions, and 200 μl of eluted DNA—to be used in the quantitative PCR (qPCR) assays—was stored at– 80°C.

For the construction of qPCR standard curves, DNA was extracted from overnight cultures of *S*. *agalactiae* LMG 14694^T^ on TSA + 5% sheep blood, *E*. *coli* ATCC 25922 grown on TSA + 5% sheep blood, and *C*. *albicans* ATCC 90028 grown on Sabouraud agar (all BD). All growth was harvested from the plate and resuspended in 1 ml of saline. DNA of this suspension was extracted using the High Pure PCR Template Preparation Kit (Roche Applied Science, Basel, Switzerland) according to the manufacturer’s instructions.

For capsular genotyping of GBS, 1 ml of inoculated Lim Broth medium (see Microbiological culturing) was used for DNA extraction using the High Pure PCR Template Preparation Kit (Roche), according to the the manufacturer’s instructions.

### *Streptococcus agalactiae* qPCR

To detect *S*. *agalactiae* in vaginal DNA extracts, a *S*. *agalactiae* specific qPCR was carried out, using primers previously described [23]. The qPCR reactions for *S*. *agalactiae* were performed in a final volume of 10 μl, containing 5 μl of LightCycler 480® SYBR Green I Master (Roche), 0.5 μM of both forward primer Sip1 (5’-ATCCTGAGACAACACTGACA-3’) and reverse primer Sip2 (5’-TTGCTGGTGTTTCTATTTTCA-3’), 0.3 μM of probe (5’- 6-FAM–ATCAGAAGAGTCATACTGCCACTTC–TAMRA-3’) (Eurogentec, Liège, Belgium) and 2 μl of DNA extract or 2 μl of HPLC water (as negative template control). Cycling conditions were as follows: 95°C for 5 min; 40 cycles of 95°C for 10 s, 58°C for 15 s and 72°C for 20 s. For the standard series, DNA concentration of the extract of *S*. *agalactiae* LMG 14694^T^ was determined using the Qubit® Fluorometer (Invitrogen, Auckland, New Zealand) and the genomic concentration was calculated based on the GC% content and genome size of the type strain. A tenfold dilution standard series of *S*. *agalactiae* LMG 14694^T^ DNA was prepared by dilution of the DNA stock in HPLC grade water. All standard tenfold dilution series and samples were run in duplicate. Amplification, detection and quantification were carried out using the LightCycler480® platform and the LightCycler® 480 Software Version 1.5 (Roche).

### *Escherichia coli* qPCR

To detect *E*. *coli* in vaginal DNA extracts, an *E*. *coli* specific qPCR was carried out, using primers targeting the β-glucuronidase encoding gene uidA, previously described [24]. The qPCR reactions were performed in a final volume of 10 μl, containing 5 μl of LightCycler 480® SYBR Green I Master (Roche), 0.3 μM of both forward primer EcoliFW (5’-CAACGAACTGAACTGGCAGA-3’) and reverse primer EcoliRV (5’- CATTACGCTGCGATGGAT -3’) (Eurogentec) and 2 μl of DNA extract or 2 μl of HPLC water (as negative template control). Cycling conditions were as follows: 50°C for 2 min, 95°C for 10 min; 40 cycles of 95°C for 15 s and 60°C for 1 min. A standard series (using *E*. *coli* ATCC 25922 grown on TSA + 5% sheep blood (BD)), was constructed as described for *S*. *agalactiae*.

### *Candida albicans* qPCR

To detect *C*. *albicans* in vaginal DNA extracts, a *C*. *albicans* specific qPCR was carried out, using primers targeting the ITS-1 gene (adapted from [25]). The qPCR reactions were performed in a final volume of 10 μl, containing 5 μl of LightCycler 480® SYBR Green I Master (Roche), 0.3 μM of both forward primer CA_FW (5’-CAACGAACTGAACTGGCAGA-3’) and reverse primer CA_RV (5’- CATTACGCTGCGATGGAT -3’) (Eurogentec) and 2 μl of DNA extract or 2 μl of HPLC water (as negative template control). Cycling conditions were as follows: 50°C for 2 min, 95°C for 10 min; 40 cycles of 95°C for 15 s and 60°C for 1 min. A standard series (using *C*. *albicans* ATCC 90028 grown on Sabouraud agar (BD)), was constructed as described for *S*. *agalactiae*.

#### Microbiological culturing

At the local laboratories, the Amies swab was inoculated on in-house TMB^plus^ plates (a medium supporting growth of anaerobes and allowing assessment of hydrogen peroxide production of strains) [26], after which the plates were incubated anaerobically as described previously [27]. After 48–72 h, depending on the growth, all biological material of the culture plate was harvested using sterile cotton swabs and stored in cryovials with 1 ml of tryptic soy broth + 5% glycerol at– 80°C until shipment. After shipment to the ITM, bacteria from the cryovial were inoculated in commercial Lim Broth medium (BD)–a selective enrichment medium for GBS–according to the manufacturer’s instructions (5% CO_2_ at 35°C for 24 hours). The latter procedure was performed only for women found to be positive for vaginal GBS carriage by means of qPCR. DNA extracts of inoculated Lim Broth medium was used for direct molecular capsular typing of GBS.

### *S*. *agalactiae* molecular capsular typing

To determine the GBS serotype, we used a flowchart described by [28], based on the multiplex PCRs with primers as described by Poyart and co-workers and Imperi and co-workers [29, 30]. The multiplex PCRs were performed directly on DNA extracted from the inoculated Lim Broth medium. The reactions were performed in a final reaction mixture of 20 μl, containing 10 μl of FastStart PCR Master Mix (Roche), 0.2 μM of each primer, and 2 μl of DNA template. Using a Veriti 96-well thermal cycler (Applied Biosystems, Foster City, CA), the following PCR program was run: 94°C for 5 min, 3 cycles of 45 s at 94°C, 2 min at 50°C, 1 min at 72°C, and 30 cycles of 20 s at 94°C, 1 min at 50°C and 1 min at 72°C, with a final extension at 72°C for 7 min. PCR amplification products were visualised under UV light after electrophoresis on 1% agarose gels (30 minutes at 10 V/cm) and staining with ethidiumbromide. Twenty five control strains (covering all GBS serotypes and provided by the Belgian *Streptococcus agalactiae* reference center (Dr. Pierette Melin, University of Liège, Belgium)) were used as a positive control.

### Physiological parameters

Vaginal pH was measured during the speculum examination by pressing commercial pH strips (pH Fix 3.6–6.1, Machery-Nagel) against the vaginal wall.

Detection of prostate-specific antigen (PSA), a marker for sexual intercourse within the past 24 hours [31] in vaginal swab fluid was performed using a chromatographic immune assay (the Seratec® PSA SemiQuant Cassette Test, Seratec, Gottingen, Germany) according to the manufacturer’s instructions. Pregnancy was assessed by testing urine with a rapid hCG test (QuickVue One-Step hCG Test (Kigali, Johannesburg) or Unimed First Sign hCG test (Mombasa)). Leucocytes and erythrocytes in urine were detected using dipsticks according to the manufacturer’s instructions (Siemens Multistix 10 sg in Kigali, Mission® urinalysis strips in Mombasa, and Neotest 4 Urine Dipstick in Johannesburg).

### Statistical analysis

Data were analyzed with SPSS software version 22 (SPSS Inc.). Prevalences were reported with their 95% confidence interval. Outcomes for this analysis were vaginal GBS carriage and vaginal *E*. *coli* carriage, as determined by a positive qPCR.

Independent variables considered were study site, sociodemographic characteristics, reproductive health characteristics, sexual behavioural factors, vaginal practices characteristics, cervicovaginal signs and symptoms and microbiological characteristics. Variables were analyzed using logistic regression in univariable and multivariable ways, with p-values < 0.05 indicating significance. In order not to overfit our multivariable models, variables were restricted in proportion to the number of cases positive for GBS and *E*. *coli*, i.e. maximum one degree of freedom per 10 cases [32]. Variables included in the models were selected as follows [33]: firstly, only variables found to be significantly associated with GBS or *E*. *coli* carriage in univariable analysis were considered for inclusion the multivariate GBS or *E*. *coli* model, respectively. Subsequently, of correlated variables (e.g. ‘having had recent vaginal intercourse’ and a positive PSA test), only one was kept for further consideration to avoid collinearity. The final selection of variables was based on literature and clinical expertise/relevance. The multivariable models were controlled for possible confounding variables and were validated with bootstrap analysis.

### Ethics statement

Written information and consent forms in the local language were provided to the women or to the Legally Authorized Representatives for their review. After the interview, the participants were asked to express their willingness to participate in the study by signing (or thumb-printing in case they were illiterate) the consent form. In case they were of minor age (age below 18 in Kenya and SA, and below 21 in Rwanda), also the parents or guardians were asked to give consent. The study was approved by the Kenyatta National Hospital Ethical Review Committee, Kenya; the Human Research Ethics Committee (Medical), University of the Witwatersrand, SA; the Rwanda National Ethics Committee, Rwanda; the Institutional Review Boards of the Institute of Tropical Medicine in Antwerp, of Ghent University, and of the University Teaching Hospital in Antwerp, Belgium. In addition, the study was approved by the National Council on Science and Technology in Kenya, and the National AIDS Control Commission in Rwanda. The study is registered at the Trial Registration at the National Health Research Ethics Council South Africa (DOH2709103223) [19].

## Results

### Vaginal GBS and *E*. *coli* carriage and GBS serotype distribution

Of the 430 women enrolled in the study, 424 and 421 vaginal swab DNA extracts were analysed for the presence of GBS and *E*. *coli*, respectively. The vaginal GBS and *E*. *coli* carriage rates in the different study groups are presented in Table 1.

**Fig 1:**
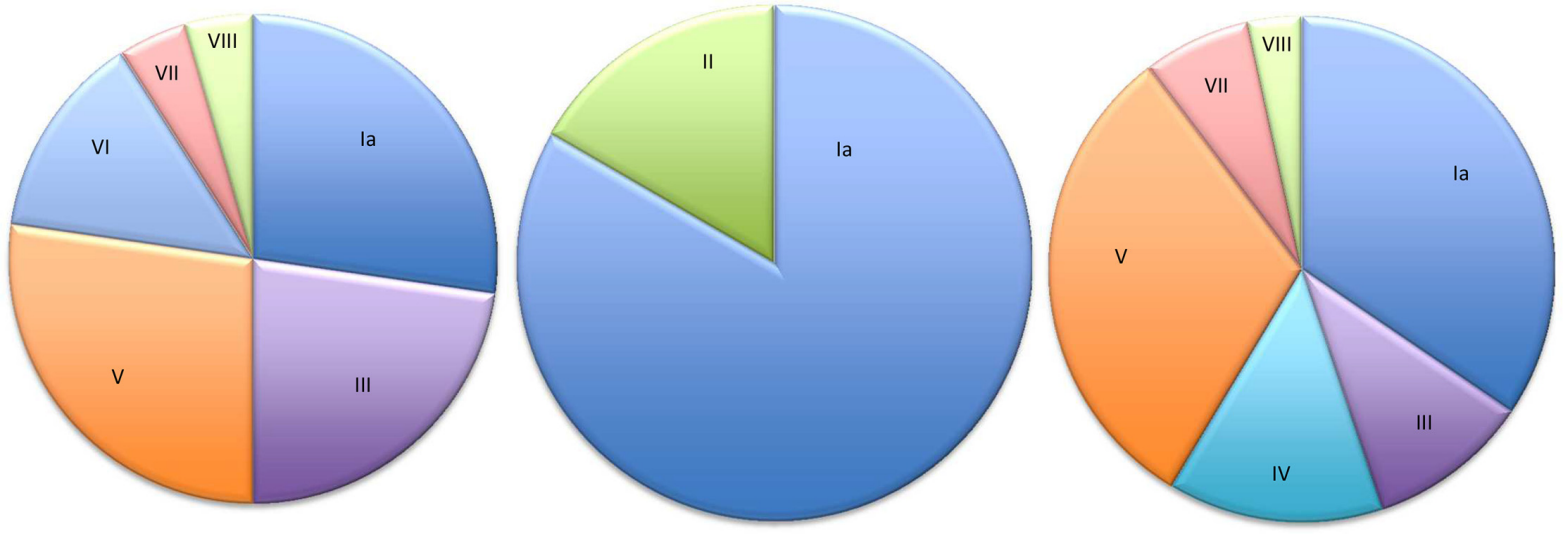
Distribution of GBS capsular serotypes. Left, Kenya (n = 22); middle, Rwanda (n = 6); right, South Africa (n = 29).

The GBS serotype distribution is presented in Table 2 and Fig 1. For 12 GBS carriers, the serotype could not be determined because samples were no longer available. Serotype distribution was largely comparable between sites. The most prevalent serotypes were Ia (27.3%), V (27.3%), and III (22.7%) in Kenya; Ia (34.5%), V (31.0%), and IV (13.8%) in SA; and Ia (83.3%) and II (16.7%) in Rwanda.

**Table 2:** Studies reporting GBS serotype distribution of (recto)vaginal isolates in SSA.

Country	Year	Population	Ia	Ib	II	III	IV	V	VI	VII	VIII	IX	NT	Reference
The Gambia	1994	P	19		28	6	3	38						[34]^$^
Malawi	2011	P, HIV+, HIV-	18.2	6.2	10.3	39.0	0.3	23.9	0.8		0.8		1.5	[35]
SA	2011	P	30.1	6.7	11.3	37.3	3.7	10.2						[36]
SA	2014	P	36.2–41.4	3.5–4.6	7.2–7.5	31.3–34.9	2.0–4.0	10.3–15.6				0.0–3.3		[37]
**Kenya**	**2015**	**P, NP**	**27.3**			**22.7**		**27.3**	**13.6**	**4.5**	**4.5**			**This study**
**Rwanda**	**2015**	**NP**	**83.3**		**16.7**									**This study**
**SA**	**2015**	**P, NP**	**34.5**			**10.3**	**13.8**	**31.0**		**6.9**	**3.4**			**This study**
Europe	2010	N/A	18.2	12.4	14.4	28.1	3.7	14.9	0.6	0.6	0.6			[38]^£^
US	2010	N/A	26.8	8.1	10.9	24.8	1.0	15.0	0.3	0.0	0.2			[38]^£^

P, pregnant; NP, non-pregnant

^$^determined serotypes I-VI (no differentiation between Ia and Ib); N/A, not applicable (review)

^£^data from meta-analysis but excluding isolates from non-sterile sites and from neonates were excluded.

### Univariable and multivariable analyses

Tables 3 and 4 present the univariable associations of the sociodemographics, sexual behavior, vaginal practices, cervicovaginal signs and symptoms, and microbiological characteristics with vaginal GBS and *E*. *coli* carriage, respectively. Because of the low prevalence, CT, NG, TV, and syphilis were not considered for further analysis.

**Table 3:** Sociodemographic characteristics, reproductive health, sexual behavior, vaginal practices, vaginal signs & symptoms, and microbiological associations with vaginal GBS carriage (univariable analysis).

	n	GBS+ n (%)	Crude OR (95% CI)	p-value*
	424	69 (16.3)		
**Sociodemographic characteristics**				
**City (Country)**				
Mombasa (Kenya)	165	27 (16.4)	0.89 (0.51–1.53)	0.665
Kigali (Rwanda)	60	6 (10.0)	0.50 (0.20–1.26)	0.142
Johannesburg (SA)	199	36 (18.1)	1	-
**Age (years)**				
<18	58	1 (1.7)	0.07 (0.01–0.50)	**0.008**
18–24	148	23 (15.5)	0.71 (0.41–1.23)	0.219
>24	218	45 (20.6)	1	-
**Educational level**				
Higher educational level^£^	189	39 (20.6)	1	-
Lower educational level^££^	235	30 (12.8)	0.56 (0.33–0.95)	**0.030**
**Marital status**				
Never married	242	34 (14.0)	1	-
Married	148	30 (20.2)	1.55 (0.91–2.67)	0.109
Separated/divorced/widowed	34	5 (14.7)	1.06 (0.38–2.91)	0.918
**Socio-economic status**^**#**^				
Low	106	17 (13.0)	1	-
Medium	163	31 (19.0)	1.23 (0.64–2.36)	0.533
High	155	21 (13.5)	0.82 (0.41–1.64)	0.576
**Reproductive health**				
**Pregnant**				
No	366	62 (16.9)	1	-
Yes	58	7 (12.1)	0.67 (0.29–1.55)	0.353
**Parity**				
0	149	18 (12.1)	1	-
1–2	211	42 (19.9)	1.81 (1.00–3.29)	0.052
>2	64	9 (14.1)	1.19 (0.50–2.81)	0.690
**Gravity**				
0	118	14 (11.9)	1	-
1–2	210	38 (18.1)	1.64 (0.85–3.17)	0.141
>2	96	17 (17.7)	1.60 (0.74–3.44)	0.230
**Regular cycle**				
Yes	256	42 (16.4)	1	-
No/unknown	168	27 (16.1)	0.98 (0.58–1.66)	0.927
**Menstrual cycle**				
No cycle	194	33 (17.0)	1	-
With cycle	230	36 (15.7)	0.91 (0.54–1.51)	0.706
**Contraceptive**				
None	73	15 (20.5)	1	-
Condom only	108	10 (9.3)	0.40 (0.17–0.94)	**0.035**
Others (hormones/IUD/sterilisation/pregnant)	243	44 (18.1)	0.86 (0.44–1.65)	0.639
**Currently breastfeeding**				
No	390	64 (16.4)	1	-
Yes	34	5 (14.7)	0.88 (0.33–2.36)	0.796
**Sexual behaviour**				
**Age at first sexual encounter (years)**				
<16	80	8 (10.0)	1	-
16–18	197	36 (18.3)	2.01 (0.89–4.5)	0.093
19–21	104	16 (15.4)	1.64 (0.66–4.04)	0.286
>21	43	9 (20.9)	2.38 (0.85–6.71)	0.101
**Sexually active (last 3 months)**				
No	55	4 (7.3)	1	-
Yes	369	65 (17.6)	2.73 (0.95–7.81)	0.062
**Condom use (at last sexual encounter)**^**@**^				
No	229	47 (20,5)	1.75 (0.94–3.16)	0.050
Yes	140	18 (12.9)	1	-
**Lifetime n° of sex partners**				
1	112	13 (11.6)	1	-
2–3	186	32 (17.2)	1.58 (0.79–3.16)	0,194
> 3	126	24 (19.0)	1.79 (0.86–3.71)	0,117
**N° of sex partners in the last 3 months**				
0	25	0 (0.0)	1	-
> = 1	399	69 (17.3)	N/A	**0.010**
**Recent vaginal sex**^**%**^				
No	343	49 (14.3)	1	-
Yes	81	20 (24.7)	2.73 (0.95–7.81)	**0.024**
**Sexual risk taking**^**€**^				
Low	167	25 (15.0)	1	-
Medium	155	30 (19.4)	1.36 (0.76–2.44)	0.297
High	102	14 (13.7)	0.90 (0.45–1.83)	0.779
**Estimated frequency of sexual encounters in last 3 months**^**@**^^**,**^ ^**&**^				
0	55	4 (7.3)	1	-
< 10 times	137	18 (13.1)	1.93 (0.62–5.98)	0.255
11–30 times	129	24 (18.6)	2.91 (0.96–8.84)	0.059
> 30 times	98	23 (23.5)	3.91 (1.27–11.98)	**0.017**
**HIV status partner**^**@**^^**,**^				
HIV positive	38	3 (7.9)	1	-
HIV negative	250	52 (20.8)	3.06 (0.91–10.36)	0.072
Unknown	79	10 (12.6)	1.69 (0.44–6.54)	0.447
**Estimated frequency of unprotected sex in last 3 months**				
No sexual contacts	55	4 (7.3)	1	-
Never unprotected	104	12 (11.5)	1.66 (0.51–5.42)	0.399
< 10 times	88	14 (15.9)	2.41 (0.75–7.75)	0.139
> = 10 times	177	39 (22.0)	3.60 (1.23–10.59)	**0.020**
**New partner (within 3 months)**				
No	378	60 (15.9)	1	-
Yes	46	9 (19.6)	1.29 (0.59–2.81)	0.523
**Circumcision status partner**^**@**^^**,**^				
Circumcised	240	38 (15.8)	1	-
No/don’t know Not circumcised/don’t know	129	27 (20.9)	1.41 (0.81–2.43)	0.222
**Female sex worker**				
Yes	30	6 (20.0)	1	-
No	394	64 (16.2)	1.03 (0.38–2.97)	0.952
**Vaginal practices**				
**Washing inside the vagina when bathing**				
No	176	15 (8.5)	1	-
Yes	248	54 (21.8)	2.99 (1.63–5.49)	**<0.001**
**Drying the vagina before sex**				
Yes	10	4 (40.0)	1	-
No	414	65 (15.7)	3.58 (0.98–13.04)	0.053
**Washed inside the vagina recently (morning or evening before study visit)**				
No	231	28 (12.1)	1	-
Yes	193	41 (21.2)	1.96 (1.16–3.30)	**0.012**
**Products to wash/clean/dry/tighten the vagina**				
None	153	13 (8.5)	1	-
Water/fingers only or water/soap	211	44 (20.9)	2.84 (1.47–5.48)	**0.002**
Cloth	48	9 (18.8)	2.49 (0.99–6.24)	0.053
Lemon juice/detergents	12	3 (25.0)	3.59 (0.86–14.92)	0.079
**Cleaning the vagina after sexual intercourse**				
No	227	28 (12.3)	1	-
Yes	197	41 (20.8)	1.87 (1.11–3.16)	**0.019**
**Cervicovaginal signs and symptoms**				
**Ectopy**^**¶**^				
No	226	34 (15.0)	1	-
Yes	197	34 (17.3)	1.18 (0.70–1.98)	0.536
**Degree of ectopy**^**¶**^				
Absent	226	34 (15.0)	1	-
Small	53	8 (15.1)	1.00 (0.44–2.32)	0.993
Moderate	139	24 (17.3)	1.18 (0.67–2.09)	0.573
Large	5	2 (40.0)	3.77 (0.61–23.4)	0.155
**Colposcopic findings**^**§**^^**,**^^**¶**^				
No	380	59 (15.5)	1	-
Yes	43	10 (23.3)	1.65 (0.77–3.53)	0.197
**Cervical mucus**				
No	270	39 (14.4)	1	-
Mild to moderate	140	28 (20.0)	1.48 (0.87–2.53)	0.151
Abundant	14	2 (14.3)	0.99 (0.21–4.58)	0.987
**Reported abnormal discharge**				
No	399	64 (16.0%)	1	-
Yes	25	5 (20%)	1.31 (0.47–3.61)	0.604
**Vaginal discharge on speculum**				
No	332	52 (15.7)	1	-
Yes	92	17 (18.5)	0.82 (0.45–1.50)	0.518
**Vaginal epithelial abnormalities**				
No	419	67 (16.0)	1	-
Yes	5	2 (40.0)	3.50 (0.57–21.36)	0.174
**Cervical epithelial abnormalities**				
No	379	64 (16.9)	1	-
Yes	45	5 (11.1)	0.62 (0.23–1.62)	0.325
**Red blood cells in urine**				
No	358	58 (16.2)	1	-
Yes	66	11 (16.7)	1.03 (0.51–2.10)	0.925
**White blood cells in urine**				
No	328	56 (17.1)	1	-
Yes	96	13 (13.5)	0.76 (0.40–1.46)	0.411
**Microbiological factors**				
**BV visit 1 (Amsel criteria)**				
No BV	346	61 (17.6)	1	-
BV	78	8 (10.3)	0.53 (0.24–1.17)	0.116
**BV (Nugent)** ^**¶**^				
No BV (Nugent 0–3)	217	37 (17.1)	1	-
Intermediate (Nugent 4–6)	29	6 (20.7)	1.30 (0.60–2.83)	0.507
BV (Nugent 7–10)	137	13 (9.5)	0.29 (0.12–0.70)	**0.006**
**Reproductive tract infection (RTI)**				
No RTI	352	60 (17.0)	1	-
1 or more RTI	60	8 (13.3)	0.75 (0.34–1.66)	0.475
>1 RTI	12	1 (8.3)	0.44 (0.06–3.50)	0.439
**Syphilis**				
No	415	69 (16.6)	1	-
Yes	9	0 (0.0)	N/A	**0.001**
***Chlamydia trachomatis***				
No	382	62	1	-
Yes	42	7	1.03 (0.44–2.43)	0.942
***Neisseria gonorrhoeae***				
No	415	69 (16.6)	1	-
Yes	9	0 (0.0)	N/A	**0.001**
***Trichomonas vaginalis***				
No	390	66 (16.9)	1	-
Yes	26	3 (11.5)	0.64 (0.19–2.20)	0.478
***Candida albicans* (qPCR)**				
No	375	50 (13.3)	1	-
Yes	46	17 (37.0)	3.81 (1.95–7.44)	**<0.001**
***Escherichia coli* (qPCR)**				
No	354	89 (25.1)	1	-
Yes	67	29 (43.3)	2.27 (1.33–3.90)	**0.003**
**HSV-2 serology**				
No	276	42 (15.2%)	1	-
Yes	147	27 (18.4%)	1.25 (0.74–2.13)	0.404
**Vaginal pH**				
< 4.4	126	15 (11.9)	1	-
4.4–5.3	240	42 (17.5)	1.57 (0.83–2.96)	0.163
5.4 and more	58	12 (20.7)	1.93 (0.84–4.44)	0.122
**PSA present**				
No	233	31 (13.3)	1	-
Yes	181	38 (21.0)	1.73 (1.03–2.91)	**0.039**
**Systemic antibiotics visit 1**				
No	341	62 (18.1)	1	-
Yes	83	7 (8.4)	0.41 (0.18–0.94)	**0.036**
**Systemic antibiotics screening visit**				
No	391	68 (17.4)	1	-
Yes	33	1 (3.0)	0.15 (0.02–1.11)	0.063

^*^bold: significant at the 5% level

^£^completed secondary school, or post-secondary school

^££^Primary school (completed or not), secondary school but not completed

^#^Socio-economic-status was constructed from total income, type of housing, type of toilet

^@^with partners within three months prior to enrolment

^&^missing data for 5

^%^sex morning or evening before visit &

^€^low risk: 1 or no partners in last year and did not have any partner (in the last 3 months) with multiple partners and age first sex at least 15 years; medium risk: 2 partners last year or had at least one sexual partner (in the last 3 months) who had multiple partners; high risk: sex worker or at least 3 partners last year or at had at least one sexual partner with HIV in the last 3 months or age first sex less than 15 years; N/A, no odds ratio due to no cases in one category

^¶^data missing for 1 (ectopy, colposcopic findings, HSV-2 serology), 3 (*C*. *albicans*, *E*. *coli*), 8 (*T*. *vaginalis*), 41 (BV Nugent, unreadable slides)

^§^petechiae (6 GBS cases/20), abrasion (2 GBS cases/5), erythema (1 GBS case/10), laceration (1 GBS case/4); BV, bacterial vaginosis; IUD, intrauterine device.

**Table 4:** Sociodemographic characteristics, reproductive health, sexual behavior, vaginal practices, vaginal signs & symptoms, and microbiological associations with vaginal *E*. *coli* carriage (univariable analysis).

	n	*E*. *coli* + n (%)	Crude OR (95% CI)	p-value*
	421	118 (28.0)		
**Sociodemographic characteristics**				
**City (Country)**				
Mombasa (Kenya)	164	39 (23.6)	0.87 (0.54–1.41)	0.569
Kigali (Rwanda)	60	27 (45.0)	2.28 (1.25–4.15)	**0.007**
Johannesburg (SA)	197	52 (26.5)	1	-
**Age (years)**				
<18	58	12 (20.7)	0.73 (0.36–1.47)	0.376
18–24	147	49 (33.3)	1.40 (0.88–2.20)	0.154
>24	216	57 (26.4)	1	-
**Educational level**				
Higher educational level^£^	187	56 (29.9)	1	-
Lower educational level^££^	234	62 (26.5)	0.84 (0.55–1.29)	0.434
**Marital status**				
Never married	240	71 (29.6)	1	-
Married	147	37 (25.2)	0.80 (0.50–1.27)	0.348
Separated/divorced/widowed	34	10 (29.4)	0.99 (0.45–2.18)	0.984
**Socio-economic status**^**#**^				
Low	106	27 (25.5)	1	-
Medium	163	46 (28.2)	1.15 (0.66–2.00)	0.620
High	152	45 (29.6)	1.23 (0.70–2.15)	0.467
**Reproductive health**				
**Pregnant**				
No	363	104 (28.7)	1	-
Yes	58	14 (24.1)	0.79 (0.42–1.51)	0.478
**Parity**				
0	149	35 (23.5)	1	-
1–2	209	62 (29.7)	1.37 (0.85–2.22)	0.196
>2	63	21 (33.3)	1.63 (0.85–3.11)	0.139
**Gravity**				
0	118	25 (21.2)	1	-
1–2	208	63 (30.3)	1.62 (0.95–2.75)	0.077
>2	95	30 (31.6)	1.72 (0.93–3.19)	0.087
**Regular cycle**				
Yes	254	65 (25.6)	1	-
No/unknown	167	54 (33.3)	1.42 (0.92–2.18)	0.111
**Menstrual cycle**				
No cycle	194	58 (29.9)	1	-
With cycle	227	60 (26.4)	0.84 (0.55–1.29)	0.430
**Contraceptive**				
None	72	20 (27.8)	1	-
Condom only	106	28 (26.4)	0.93 (0.48–1.83)	0.841
Others (hormones/IUD/sterilisation/pregnant)	243	70 (28.8)	1.05 (0.59–1.89)	0.865
**Currently breastfeeding**				
No	387	106 (27.4)	1	-
Yes	34	12 (35.3)	1.45 (0.69–3.03)	0.327
**Sexual behaviour**				
**Age at first sexual encounter (years)**				
<16	79	29 (36.7)	1	-
16–18	197	52 (26.4)	0.62 (0.35–1.08)	0.090
19–21	103	26 (25.2)	0.58 (0.31–1.10)	0.096
>21	42	11 (26.2)	0.61 (0.27–1.40)	0.224
**Sexually active (last 3 months)**				
No	55	16 (29.1)	1	-
Yes	366	102 (27.9)	0.94 (0.50–1.76)	0.851
**Condom use (at last sexual encounter)**				
No	283	68 (24.0)	1.80 (1.16–2.79)	**0.009**
Yes	138	50 (36.2)	1	-
**Lifetime n° of sex partners**				
1	110	28 (25.5)	1	-
2–3	185	42 (22.7)	0.86 (0.50–1.49)	0.591
> 3	126	48 (38.1)	1.80 (1.03–3.15)	**0.039**
**N° of sex partners in the last 3 months**				
0	25	6 (24.0)	1	-
> = 1	396	112 (28.3)	1.25 (0.49–3.21)	0.644
**Recent vaginal sex**^**%**^				
No	340	91 (26.8)	1	-
Yes	81	27 (33.3)	1.37 (0.81–2.30)	0.238
**Sexual risk taking**^**€**^				
Low	167	44 (26.3)	1	-
Medium	152	37 (22.4)	0.90 (0.54–1.49)	0.681
High	102	37 (36.3)	1.59 (0.94–2.71)	0.086
**Estimated frequency of sexual encounters in last 3 months**^**@**^^**,**^ ^**&**^				
0	55	16 (29.1)	1	-
< 10 times	135	31 (23.0)	0.73 (0.36–1.47)	0.376
11–30 times	128	43 (33.6)	1.23 (0.62–2.45)	0.550
> 30 times	98	27 (27.6)	0.93 (0.45–1.93)	0.839
**HIV status partner**^**@**^				
HIV positive	38	11 (28.9)	1	-
HIV negative	247	63 (25.5)	0.84 (0.39–1.79)	0.653
Unknown	79	28 (35.4)	1.35 (0.58–3.12)	0.486
**Estimated frequency of unprotected sex in last 3 months**				
No sexual contacts	55	16 (29.1)	1	-
Never unprotected	102	34 (33.3)	1.22 (0.60–2.49)	0.586
< 10 times	87	19 (21.8)	0.68 (0.31–1.48)	0.330
> = 10 times	177	49 (27.7)	0.93 (0.48–1.82)	0.839
**New partner (within 3 months)**				
No	375	96 (25.6)	1	-
Yes	46	22 (47.8)	2,66 (1.43–4.97)	**0.002**
**Circumcision status partner**^**@**^				
Circumcised	238	62 (26.1)	1	-
Not circumcised/don’t know	128	40 (31.3)	1.29 (0.80–2.07)	0.291
**Female sex worker**				
No	391	97 (24.8)	1	-
Yes	30	21 (70.0)	7.07 (3.13–15.96)	**<0.001**
**Vaginal practices**				
**Washing inside the vagina when bathing**				
No	175	44 (25.1)	1	-
Yes	246	74 (30.1)	1.28 (0.83–1.98)	0.267
**Drying the vagina before sex**				
No	411	114 (27.3)	1	-
Yes	10	4 (40.0)	1.74 (0.48–6.27)	0.399
**Washed inside the vagina recently (morning or evening before study visit)**				
No	229	64 (27.9)	1	-
Yes	192	54 (28.1)	1.01 (0.66–1.55)	0.968
**Products to wash/clean/dry/tighten the vagina**				
None	153	40 (26.1)	1	-
Water/fingers only or water/soap	210	59 (28.1)	1.10 (0.69–1.77)	0.680
Cloth	46	16 (34.8)	1.51 (0.74–3.05)	0.255
Lemon juice/detergents	12	3 (25.0)	0.94 (0.24–3.65)	0.931
**Cleaning the vagina after sexual intercourse**				
No	226	61 (27.0)	1	-
Yes	195	57 (29.2)	1.12 (0.73–1.71)	0.610
**Cervicovaginal signs and symptoms**				
**Ectopy**^**¶**^				
No	224	49 (21.9)	1	-
Yes	196	69 (35.2)	1.94 (1.26–2.99)	**0.003**
**Degree of ectopy**^**¶**^				
Absent	224	49 (21.9)	1	-
Small	52	22 (42.3)	2.62 (1.39–4.94)	**0.003**
Moderate	139	46 (33.1)	1.77 (1.10–2.84)	**0.019**
Large	5	1 (20.0)	0.89 (0.10–8.17)	0.920
**Colposcopic findings**^**§**^^**,**^ ^**¶**^				
No	377	102 (27.1)	1	-
Yes	43	16 (37.2)	1.60 (0.83–3.09)	0.163
**Cervical mucus**				
No	269	67 (24.9)	1	-
Mild to moderate	138	47 (34.1)	1.56 (1.00–2.44)	0.052
Abundant	14	4 (28.6)	1.21 (0.37–3.97)	0.758
**Reported abnormal discharge**				
No	396	112 (28.3)	1	-
Yes	25	6 (24.0)	0.80 (0.31–2.06)	0.644
**Vaginal discharge on speculum**				
No	331	85 (25.7)	1	-
Yes	90	33 (36.7)	1.68 (1.02–2.75)	**0.041**
**Vaginal epithelial abnormalities**				
No	416	116 (27.9)	1	-
Yes	5	2 (40.0)	1.72 (0.28–10.45)	0.554
**Cervical epithelial abnormalities**				
No	377	105 (27.9)	1	-
Yes	44	13 (29.5)	1.09 (0.55–2.16)	0.813
**Red blood cells in urine**				
No	355	101 (28.5)	1	-
Yes	66	17 (25.8)	0.87 (0.48–1.59)	0.655
**White blood cells in urine**				
No	325	81 (24.9)	1	-
Yes	96	37 (38.5)	1.89 (1.17–3.06)	**0.010**
**Microbiological factors**				
**BV visit 1 (Amsel criteria)**				
No BV	344	99 (28.8)	1	-
BV	77	19 (24.7)	0.81 (0.46–1.43)	0.469
**BV visit 1 (Nugent)** ^**¶**^				
No BV (Nugent 0–3)	217	60 (27.6)	1	-
Intermediate (Nugent 4–6)	29	15 (51.7)	2.80 (1.28–6.16)	**0.010**
BV (Nugent 7–10)	137	33 (24.1)	0.83 (0.51–1.36)	0.459
**GBS**				
No	354	89 (25.1)	1	-
Yes	67	29 (43.3)	2.27 (1.33–3.90)	**0.003**
**Reproductive tract infection (RTI)**				
No RTI	349	96 (27.5)	1	-
1 or more RTI	60	19 (31.7)	1.22 (0.68–2.21)	0.508
>1 RTI	12	3 (25.0)	0.88 (0.23–3.31)	0.848
**Syphilis**				
No	412	117 (28.4)	1	-
Yes	9	1 (11.1)	0.32 (0.04–2.55)	0.279
***Chlamydia trachomatis***				
No	379	107 (28.2)	1	-
Yes	42	11 (26.2)	0.90 (0.44–1.86)	0.780
***Neisseria gonorrhoeae***				
No	412	114 (27.7)	1	-
Yes	9	4 (44.4)	2.09 (0.55–7.93)	0.278
***Trichomonas vaginalis***^**¶**^				
No	387	105 (27.1)	1	-
Yes	26	11 (42.3)	1.97 (0.88–4.43)	0.101
***Candida albicans* (qPCR)**				
No	375	102 (27.2)	1	-
Yes	46	16 (34.8)	1.43 (0.75–2.73)	0.282
**HSV-2 serology**^**¶**^				
No	274	80 (29.2)	1	-
Yes	146	38 (26.0)	0.85 (0.54–1.34)	0.492
**Vaginal pH**				
< 4.4	125	34 (27.2)	1	-
4.4–5.3	237	65 (27.4)	1.01 (0.62–1.65)	0.963
5.4 and more	58	19 (32.8)	1.30 (0.66–2.56)	0.441
**PSA present**				
No	231	69 (29.9)	1	-
Yes	180	48 (26.7)	0.85 (0.55–1.32)	0.475
**Systemic antibiotics visit 1**				
No	338	98 (29.0)	1	-
Yes	83	20 (24.0)	0.78 (0.45–2.36)	0.374
**Systemic antibiotics screening visit**				
No	388	111 (28.6)	1	-
Yes	33	7 (21.2)	0.67 (0.28–1.59)	0.366

^*^Bold: significant at the 5% level

^£^completed secondary school, or post-secondary school

^££^Primary school (completed or not), secondary school but not completed

^#^Socio-economic-status was constructed from total income, type of housing, type of toilet

^@^with partners within three months prior to enrolment

^&^missing data for 5

^%^sex morning or evening before visit

^€^low risk: 1 or no partners in last year and did not have any partner (in the last 3 months) with multiple partners and age first sex at least 15 years; medium risk: 2 partners last year or had at least one sexual partner (in the last 3 months) who had multiple partners; high risk: sex worker or at least 3 partners last year or at had at least one sexual partner with HIV in the last 3 months or age first sex less than 15 years; N/A, no odds ratio due to no cases in one category

^¶^data missing for 1 (ectopy, colposcopic findings, HSV-2 serology), 8 (*T*. *vaginalis*), 38 (BV Nugent, unreadable slides)

^§^petechiae (6 *E*. *coli* cases/20), abrasion (2 *E*. *coli* cases/5), erythema (3 *E*. *coli* cases/10), laceration (2 *E*. *coli* cases/4), ulcer (2 E. coli cases/6), ecchymosis (2 *E*. *coli* cases/6); BV, bacterial vaginosis.

In our final multivariable GBS model (Table 5), BV by Nugent score remained significantly negatively associated with GBS carriage (AOR, 0.43; 95% CI, 0.21–0.88; p = 0.022), and a positive association was observed for vaginal *Candida albicans* carriage (AOR, 3.25; 95% CI, 1.50–7.06; p = 0.003), vaginal *E*. *coli* carriage (AOR, 2.01; 95% CI, 1.10–3.80; p = 0.023), recent vaginal intercourse (AOR, 2.63; 95% CI, 1.35–5.15; p = 0.005), and currently washing the vagina (AOR, 2.26; 95% CI, 1.16–4.37; p = 0.016).

**Table 5:** Multivariable associations with vaginal GBS carriage.

	n	GBS+ (%)	adjusted OR (95% CI)	p-value^$^
	424	69 (16.3)		
**Recent vaginal sex**^**%**^				
No	343	49 (14.3)	1	-
Yes	81	20 (24.7)	2.63 (1.35–5.15)	**0.005**
**Washing inside the vagina**^**#**^				
No	176	15 (8.5)	1	-
Yes	248	54 (21.8)	2.26 (1.16–4.37)	**0.016**
**BV (Nugent)**^**¶**^				
No BV (Nugent 0–3)	217	37 (17.1)	1	-
Intermediate (Nugent 4–6)	29	6 (20.7)	0.93 (0.33–2.64)	0.898
BV (Nugent 7–10)	137	13 (9.5)	0.43 (0.21–0.88)	**0.022**
***Candida albicans* (qPCR)**^**¶**^				
No	375	50 (13.3)	1	-
Yes	46	17 (37.0)	3.25 (1.50–7.06)	**0.003**
***Escherichia coli*** ^**¶**^				
No	303	38 (12.5)	1	-
Yes	118	29 (24.6)	2.01 (1.10–3.80)	**0.023**

^$^bold, significant at the 5% level

^#^when having shower or bath

^%^morning or evening before study visit

^¶^data missing for 3 (*C*. *albicans*, *E*. *coli*), 41 (BV, unreadable slides).

In our multivariable *E*. *coli* model, an intermediate Nugent score remained significantly negatively associated with vaginal *E*. *coli* carriage (AOR, 2.61; 95% CI, 1.15–5.94; p = 0.023), and a positive association was observed with working as a FSW (AOR, 7.83; 95% CI, 2.88–21.30; p<0.001), vaginal GBS carriage (AOR, 2.05; 95% CI, 1.09–3.83; p = 0.025), and cervical ectopy (AOR, 1.64; 95% CI, 1.01–2.68; p = 0.046) (Table 6).

**Table 6:** Multivariable associations with vaginal *E*. *coli* carriage.

	n	*E*. *coli* + (%)	adjusted OR (95% CI)	p-value^$^
	421	118 (25.2)		
**Condom use (at last sexual encounter)**				
No	283	68 (24.0)	1.53 (0.92–2.56)	0.104
Yes	138	50 (36.2)	1.0	-
**Female sex worker**				
No	391	97 (24.8)	1	-
Yes	30	21 (70.0)	7.83 (2.88–21.30)	**<0.001**
**Ectopy** ^**¶**^				
No	224	49 (21.9)	1	-
Yes	196	69 (35.2)	1.64 (1.01–2.68)	**0.046**
**Vaginal discharge on speculum**				
No	331	85 (25.7)	1	-
Yes	90	33 (36.7)	1.63 (0.92–2.88)	0.095
**BV (Nugent)**^**¶**^				
No BV (Nugent 0–3)	217	60 (27.6)	1	-
Intermediate (Nugent 4–6)	29	15 (51.7)	2.61 (1.15–5.94)	**0.023**
BV (Nugent 7–10)	137	33 (24.1)	0.66 (0.38–1.15)	0.140
**GBS**				
No	354	89 (25.1)	1	-
Yes	67	29 (43.3)	2.05 (1.09–3.83)	**0.025**

^$^bold, significant at the 5% level

^¶^data missing for 1 (ectopy), 38 (BV, unreadable slides).

## Discussion

Group B streptococci (GBS) and *E*. *coli* account for the majority of EOS cases worldwide.

Vaginal carriage of GBS and *E*. *coli* is considered a prerequisite for GBS or *E*. *coli* transmission to the neonate in GBS EOS and *E*. *coli* EOS, respectively. However, epidemiological data of vaginal GBS and *E*. *coli* carriage, which are essential for the development and implementation of prevention strategies, are very limited in sub-Saharan Africa (SSA) [16, 17].

In this study, we aimed to present vaginal GBS and *E*. *coli* carriage rates, GBS serotype distribution and define risk factors for carriage in populations from three SSA countries.

### Vaginal GBS and *E*. *coli* carriage rates

We found a vaginal GBS carriage rate of 20.2% and 23.2% in the Kenyan and South African reference groups (adult, non-pregant, HIV-negative women at average risk of HIV), respectively. Compared to these reference groups, adolescents in our study were found to have lower GBS carriage rates: 3.6% of the Kenyan and 0% of the SA adolescents carried GBS vaginally. Other studies report conflicting associations between age and vaginal GBS carriage [39–47]. All of these studies (except for [44]) report on pregnant women. Interestingly, when we compare different age groups (< 18 years, 18–24 years, > 24 years) in our Kenyan and SA population, we see no age-group dependent GBS colonization in the pregnant women. However, we do see a statistically significant age-group dependent GBS association in the non-pregnant women, with the lowest and the highest GBS carrier rates in the youngest and the oldest age groups, respectively (Pearson Chi-Square test, data not shown).

The pregnant women in our study population had vaginal GBS carriage rates of 14.3% and 10.0% in Kenya and SA, respectively. This is lower then most other studies reporting (recto)vaginal GBS carriage rates in SSA (see Table 7). Although the CDC recommends rectovaginal sampling for detection of GBS in pregnant women, we only swabbed vaginally, to be able to study the interaction of GBS with the vaginal immune system and vaginal microbiome (to be published). This vaginal sampling may (partly) explain the lower GBS carriage rates found by us, as rectovaginal sampling has been shown to yield higher GBS recovery rates compared to vaginal sampling alone [48, 49]. Furthermore, in contrast to other studies (listed in Table 7) using culturing techniques, we used qPCR without prior enrichment step to detect GBS. Although the CDC allows PCR for the detection of GBS (albeit recommending an enrichment step), this difference with other studies probably does not account (or to a lesser extent) for the lower rates found by us, as PCR (even without an enrichment step) has been shown to be more sensitive than culture [14, 50–53]. A further difference with other studies regards the fact that the pregnant women in our study were up to 14 weeks of gestation, while the other studies listed in Table 7 sampled pregnant women at 35–37 weeks of gestation, which also may account for differences, as some authors have reported on varying GBS rates during pregnancy [45].

**Table 7:** Studies reporting (recto)vaginal GBS carriage rates in SSA.

Country	Year	n	Population	% GBS	Sample	Detection	Reference
Nigeria	1980	588	P, L	19	V	SB+C	[55]
Nigeria	1983	225	P	20	V	SB+C	[56]
Zimbabwe	1990	89	P	31	V	SB+C	[57]
Togo	1991	106	P	4	V, R	SB+C	[58]
Gambia	1994	136	P	22	V, R	SB+C	[34]
Malawi	2005	97	P	16.5	V, R	SA	[59]
Mozambique	2008	113	P	1.8	V, R	SB+C	[60]
Tanzania	2009	300	P	23.0	V, R	SB+C	[54]
Zimbabwe	2010	780	P	47, 24, 21^#^	V, R	SB+C	[61]
Malawi	2011	1840	P, HIV+ and HIV-	21.2	V, R	SB+C	[35]
South Africa	2014	661, 621, 595, 521^$^	P	33.0, 32.7, 28.7, 28.4^$^	V, R	SA	[37]
DR Congo	2015	509	P	20.2	V	SA	[62]

L, women in labor; NP, non-pregnant women; P, pregnant women; V, vaginal swab; R, rectal swab; SB+C, selective broth and culturing; SA, selective agar

^#^week 20, 26, and delivery, respectively

^$^week 20–25, week 26–30, week 31–35, and week 37+, respectively.

In the group of HIV positive women, we did not observe any GBS carriers, which is probably explained by the fact that most of the HIV positive women (26/30) received prophylactic cotrimoxazole, which is largely effective against GBS [54].

Our reference groups from Kenya and SA had vaginal *E*. *coli* carriage rates of 25.0% and 27.1%, respectively. Compared to other studies from SSA reporting vaginal carriage of *E*. *coli*, these prevalences are higher than the ones reported by Karou and coworkers (2012) and Ekwempu and coworkers (1981), lower than the ones reported by Schellenberg and coworkers (2011) and Cutland and coworkers (2012), and comparable with the prevalence reported by Sagna and coworkers (2010) (See Table 8)[63–67]. Vaginal *E*. *coli* carriage rates in Asia, Europe, North and South America appear lower. Different study populations, sampling and detection techniques might account for these differences.

**Table 8:** Studies reporting (recto)vaginal *E*. *coli* carriage rates.

Country	Year	n	Population	% E. coli	Sample	Detection	Reference
***Africa* (pooled prevalence 36.0% (2846/7912); range 9.1–46.5%)**							
Burkina Faso	2010	156	HIV+	28.4	V	C	[67]
Burkina Faso	2012	2000	S	16.7	V	C	[63]
Kenya	2011	44	HIV+, HIV-, HESN	40.1	V	cpn60	[65]
Nigeria	1981	187	L	9.1	C	C	[64]
SA	2012	1347	P, HIV+	42.3	V	C	[66]
SA	2012	3752	P, HIV-	46.5	V	C	[66]
***Asia* (pooled prevalence 5.3% (163/3072); range 0–25.8%)**							
Iraq	2011	90	S, NP	16.2	V	C	[68]
Iraq	2011	20	S, P	25.8	V	C	[68]
Iran	2014	85	S, P	18.0	V	C	[69]
Japan	2002	2575	NP, P	3.4	V	C	[70]
Pakistan	2012	100	HC	28	V	C	[71]
Pakistan	2012	100	H	6	V	C	[71]
Turkey	2007	34	IUD	14.7	V	C	[72]
Turkey	2007	34	HC	2.9	V	C	[72]
Turkey	2007	34	H	0.0	V	C	[72]
***Europe* (pooled prevalence 13.4% (670/4980); range 3.1–51.2%)**							
Croatia	2011	114	IUD	25.5	V	C	[73]
Croatia	2011	122	H	8.2	V	C	[73]
Denmark	2014	668	P	11.7	V	C	[74]
Germany	2007	166	H	16.3, 51.2, 25.9^*^	V	C	[75]
Greece	2008	1632	S	3.1	V	C	[76]
Lithuania	2012	970	P	19.9	V, R	C	[77]
Spain	2002	623	P	27.0	V	C	[78]
Spain	2011	321	P	15	E, V	C	[79]
Spain	2011	327	NP	12	E, V	C	[79]
Sweden	2008	37	H	5.4	V	C	[80]
***North America* (pooled prevalence 12.7% (430/3373); range 0–29.5%)**							
Canada	1983	495	H	12.3	V	C	[81]
US	1997	2646	P	13.0	V	C	[6]
US	2001	44	H	18.2, 9.1, 29.5, 6.8, 6.8 ^#^	V	C	[82]
US	2005	20	H	0	V	16S	[83]
US	2012	70	P	10	V	C	[84]
US	2012	35	NP	23	V	C	[84]
US	2013	47	P	2.1, 2.2, 5.6, 8.3^£^	V	C	[85]
US	2013	16	NP	6.3, 0, 12.5, 20	V	C	[85]
***South America* (pooled prevalence 19.7% (135/684); range 14.3–23.0%)**							
Argentina	2013	259	P	14.3	V	C	[86]
Chile	2009	425	S	23.0	V	C	[87]

The electronic bibliographic database PubMed was searched for articles using the search terms ‘(Escherichia) AND (coli) AND (vaginal)’ with no date or language restriction. Studies were included if the number of vaginal *E*. *coli* carriers and the total number of individuals tested were reported; studies were excluded if women were not of childbearing age. 16S, deep sequencing of the 16S rRNA gene; cpn60, deep sequencing of the cpn60 gene; C, conventional culturing and identification; E, endocervial swab; FSW, female sex workers; H, healthy women; HC, women using hormonal contraception; HESN, HIV exposed seronegative women; IUD, women using an intrauterine device as contraception; L, women in labor; NP, non-pregnant women; P, pregnant women; Q, qPCR; R, rectal swab; S, women with vaginal symptoms or clinical diagnosis of infection; V, vaginal swab

^*^pre, mid, post cycle, respectively

# visit 1at 1 month before visit 2 and 19–24 days after cycle, 1–2 days before intercourse, 8-12h after intercourse, 3–4 days after intercourse, 5–6 days after intercourse

£ <14weeks, between 14–28 weeks, >28weeks, postpartum.

Compared to the reference group in Kenya, pregnant women had a lower prevalence of vaginal *E*. *coli* carriage; compared to the reference group in SA, adolescent women had a lower prevalence of *E*. *coli* carriage. The FSW study group in Kigali had a very high prevalence of *E*. *coli* carriage, i.e. 70%, and will be discussed below (risk factors).

To our knowledge, this is the first study to determine simultaneously the vaginal GBS and *E*. *coli* carriage rates in SSA populations using qPCR, known to be more sensitive than culture-based techniques. Moreover, vaginal carriage rates of GBS in Kenya and Rwanda and *E*. *coli* in Rwanda have not yet been described.

### Risk factors for vaginal *E*. *coli* and GBS carriage

The presence of vaginal *C*. *albicans*, recent vaginal intercourse, working as a FSW, an intermediate vaginal microbiome, BV, washing the vagina and cervical ectopy were independent risk factors for vaginal GBS or *E*. *coli* colonization.

Women carrying *C*. *albicans* were 3.6 times more likely also to carry GBS. Three US based studies have shown this same significantly positive association between GBS and *Candida* or yeast [40, 88, 89].

Intravaginal practices, like e.g. cleaning inside the vagina beyond the introitus or insertion of substances into the vagina to dry or tighten the vagina, are common in Africa and are associated with adverse outcomes including increased risk for BV and for sexually transmitted infections [90]. Our model showed that washing inside the vagina was an independent risk factor for vaginal GBS carriage: women were twice as likely to be colonized with GBS compared to women not washing inside the vagina. A study by van de Wijgert and coworkers [91] showed that women using substances other than plain water to finger-clean or wipe inside the vagina had a GBS prevalence of 26.3% (n = 99), whereas women not engaging in these practices had a GBS prevalence of 14.7% (n = 70). However, their findings did not reach significance, most probably because of the smaller sample size (169 women compared to 424 women in our study).

Women who had recent vaginal sex (the morning or evening before the study visit) were more than twice as likely to carry GBS vaginally than women who did not. Accordingly, a positive PSA test was also significantly correlated with GBS carriage. GBS is generally not considered an STI, and the influence of sexual behavior on vaginal GBS carriage or acquisition is a matter of debate [92–94]. Based on published literature and our own data, we hypothesize that sexual activity might lead to a brief temporal GBS colonization of the vagina. This hypothesis is strengthened by a recent longitudinal deep-sequencing study of the vaginal microbiome, where 25 women were sampled on a daily basis over a 10 week period, revealing an average of 0.39 GBS episodes per week and an average GBS episode of 2.8 days (Fig 1 and additional file 4 in [95]), contrasting with earlier studies—where sampling occurred every 3 weeks—that report average GBS episodes of 13.7 weeks [96]. The brief colonization might explain why we and other authors find parameters such as ‘age of first sexual intercourse’ not to be associated with GBS carriage (they do not cover the recent aspect), while parameters such as ‘high frequency of intercourse during last month’ (as a consequence, a higher chance of also having had recent intercourse) do correlate. Taken together, GBS should be considered as a potentially pathogenic micro-organism that can be sexually transmitted and whose vaginal presence can be enhanced by sexual activity.

Cervical ectopy was an independent risk factor for vaginal *E*. *coli* carriage, 21.9% of women without cervical ectopy were *E*. *coli* carriers as opposed to 35.2% of women with cervical ectopy. Cervical ectopy has been associated with CT [97], HPV [98] and an increased susceptibility to HIV infection [99]. Although we could not determine the cause-effect relation of this association, it seems biologically plausible that a niche is created by the glandular columnar epithelium of women with cervical ectopy that somehow—directly or indirectly—favors the colonization by *E*. *coli*. Some studies have e.g. related cervical ectopy with a reduced cell-mediated or changed humoral immunity [21, 100].

Working as a FSW was an independent risk factor for vaginal *E*. *coli* carriage. We could not explain this by any of the sexual behavioural or other parameters presented in Table 4. In our study, none of the participants, including the female sex workers, reported having had anal intercourse during the last 3 months. These percentages probably are underestimates, since stigma associated with anal intercourse often leads to reduced reporting [101]. Furthermore, Ghanem and coworkers [102] showed that regarding anal intercourse, significantly more women reported to engage in these practices when asked by means of computer assisted self interviews compared to face-to-face interviews, which was used in our study. It is not unlikely that FSW engage more in anal intercourse, compared to the general population, and that in our FSW population, vaginal contamination with *E*. *coli* is higher by transfer of (peri)anal bacteria during anal/vaginal intercourse. Other studies from East-Africa report anal intercourse prevalences in FSW of up to 40.8% [103]. Interestingly, anal intercourse during pregnancy has been reported as a significant risk factor for neonatal *E*. *coli* colonization [77].

Besides above-mentioned risk factors for GBS or *E*. *coli* carriage, we show for the first time that colonization with GBS and *E*. *coli*, the leading causes of EOS, are positively associated.

Abovementioned risk factors can be translated and implemented into strategies that aim to reduce the maternal carriage of GBS and *E*. *coli*. First, behavioral change by advocating abstinence from sexual intercourse and vaginal washing during late pregnancy, e.g. via counseling in family planning facilities, could help to reduce the risk of maternal GBS and *E*. *coli* colonization in resource-poor settings. Second, the extension of GBS screening with screening for *C*. *albicans* and *E*. *coli*–risk factors for vaginal GBS carriage–should be further investigated. Furthermore, the screening for *E*. *coli* itself also merits further investigation because of its role as a major EOS causative agent for which currently no prevention measures are taken, nor in low-income, nor in high-income countries [104]. In this context, the presence of cervical ectopy–a risk factor for vaginal *E*. *coli* carriage–should be further investigated.

### GBS serotype distribution

As IAP is not effective against LOS and culture-based screening and administration of costly intravenous antibiotics might not be feasible in most low-income countries, an alternative and long-term solution lies in the development of effective GBS vaccines, that however would not cover for other micro-organisms causing EOS. As most GBS vaccines under development aim at eliciting protective antibodies against capsular polysaccharides, the principal difficulty in developing globally effective GBS vaccines is the existence of several serotypes with different geographical distributions. In our study, the most prevalent GBS serotypes were Ia (27.3%), V (27.3%) and III (22.7%) in Kenya; Ia (34.5%), V (31.0%), and IV (13.8%) in SA; and Ia (83.3%) and II (16.7%) in Rwanda. Only few other studies have documented vaginal GBS serotype distribution in Africa and these are largely in line with our findings (Table 4 and Fig 1).

Interestingly, compared to the low prevalences in Europe and the US, we found relatively high prevalences of serotypes IV, VI, VII–shown for the first time in sub-Saharan Africa—and VIII in the Kenyan and South African population (VI, 13.6%; VII, 4.5%; VIII 4.5% in Kenya, and IV, 13.8%; VI, 6.9%; VIII, 3.4% in SA) (Table 4). In contrast, we did not detect any serotype Ib, which has, according to a recent meta-analysis, a prevalence of 8.1% in the US and 12.4% in Europe [38].

Differences in serotype distribution between our study and other studies (Table 4) might be explained by differences in study populations, most studies listed in Table 4 typed strains isolated from pregnant women whereas most of our GBS strains were isolated from non-pregnant women. Methodological differences might also contribute. Many GBS capsular polysaccharide typing methods have been described, with the most commonly used method being a serological test, used by all studies listed in Table 4, except our study. We used a molecular capsular typing method, developed and applied by European (reference) laboratories [105, 106]. Brigtsen and coworkers (2015) compared capsular typing of 426 GBS strains by a conventional latex agglutination test with PCR, and found that a substantial proportion of the strains were non-typeable by serotyping, but typeable by genotyping, and that an agreement between serotyping and genotyping was shown in 71.1% (of the isolates that were typeable by both methods) [107]. Moreover, we used a molecular technique directly on DNA extracts from vaginal swabs (and not on DNA extracts from isolates), which could lead to the detection of certain serotypes that would not have been isolated by culture.

Currently, there are two candidate vaccines in phase II clinical trials, i.e. a trivalent vaccine targeting serotypes Ia, Ib, and III, and a conjugate vaccine targeting serotype III (www.clinicaltrials.gov). In theory, the first vaccine could cover for 50.0%, 83.3% and 44.8% of vaginal GBS cases in our Mombasa, Kigali and Johannesburg population, respectively, but only a minority of women would be protected by the conjugate vaccine (22.7%, 0% and 10.3%, respectively).

### GBS, *E*. *coli* and the vaginal microbiome

Our results show that vaginal GBS and *E*. *coli* carriage were significantly associated with disturbances of the vaginal microbiome: compared to women with a normal vaginal microbiome, women with an intermediate vaginal microbiome were 2.61 times more likely to carry *E*. *coli*, whereas women with BV were 2.33 less likely to carry GBS. The latter finding is in accordance a study of Hillier and coworkers [108], reporting a significant negative association between GBS carriage and BV by Nugent scoring, studying 7,918 pregnant women. Two other studies did not confirm these findings [94, 109]. In depth analysis of abovementioned interdependencies of GBS, *E*. *coli*, *C*. *albicans*, BV and the vaginal microbiome will be published elsewhere.

Our study was limited by the fact that we used vaginal sampling instead of rectovaginal sampling for the detection of GBS (as recommended by the CDC), which has been shown to have higher recovery rates for GBS. Furthermore, we did not use a selective broth enrichment prior to PCR, as recommended by the CDC. In our study, pregnant women were up to 14 weeks of gestation, and were not sampled at 35–37 weeks’ gestation as recommended by the CDC, which could have biased our results. Our study was further limited by the rather small sample size of our Rwanda study population.

In conclusion, vaginal GBS carriage rate and serotype distribution were similar to high-income countries, except for the higher prevalence of serotypes VI, VII and VIII. *E*. *coli* carriage rate was higher compared to high-income countries. We identified risk factors for GBS or *E*. *coli* carriage, ie. recent sexual intercourse, vaginal washing, *C*. *albicans* colonization and presence of cervical ectopy, that can be implemented in strategies to reduce maternal colonization. Immunoprophylaxis with current phase II candidate GBS vaccines would not protect the majority of women against vaginal GBS carriage in our study population. The most important causative agents of EOS, GBS and *E*. *coli*, both associated with disturbances of the vaginal microbiome, are positively associated.

## References

[1] Lawn JE, Cousens S, Zupan J, Lancet Neonatal Survival Steering T. 4 million neonatal deaths: when? Where? Why? Lancet. 2005;365(9462):891–900. 10.1016/S0140-6736(05)71048-5 . 15752534

[2] Hornik CP, Fort P, Clark RH, Watt K, Benjamin DK Jr, Smith PB, et al Early and late onset sepsis in very-low-birth-weight infants from a large group of neonatal intensive care units. Early Hum Dev. 2012;88 Suppl 2:S69–74. 10.1016/S0378-3782(12)70019-1 22633519 PMC3513766

[3] Schuchat A. Epidemiology of group B streptococcal disease in the United States: shifting paradigms. Clin Microbiol Rev. 1998;11(3):497–513. 9665980 10.1128/cmr.11.3.497 PMC88893

[4] Cutland CL, Madhi SA, Zell ER, Kuwanda L, Laque M, Groome M, et al Chlorhexidine maternal-vaginal and neonate body wipes in sepsis and vertical transmission of pathogenic bacteria in South Africa: a randomised, controlled trial. Lancet. 2009;374(9705):1909–16. 10.1016/S0140-6736(09)61339-8 . 19846212

[5] Simonsen KA, Anderson-Berry AL, Delair SF, Davies HD. Early-onset neonatal sepsis. Clin Microbiol Rev. 2014;27(1):21–47. 10.1128/CMR.00031-13 24396135 PMC3910904

[6] Krohn MA, Thwin SS, Rabe LK, Brown Z, Hillier SL. Vaginal colonization by Escherichia coli as a risk factor for very low birth weight delivery and other perinatal complications. J Infect Dis. 1997;175(3):606–10. . 9041332 10.1093/infdis/175.3.606

[7] Acosta CD, Kurinczuk JJ, Lucas DN, Tuffnell DJ, Sellers S, Knight M, et al Severe maternal sepsis in the UK, 2011–2012: a national case-control study. PLoS Med. 2014;11(7):e1001672 10.1371/journal.pmed.1001672 25003759 PMC4086731

[8] Beck S, Wojdyla D, Say L, Betran AP, Merialdi M, Requejo JH, et al The worldwide incidence of preterm birth: a systematic review of maternal mortality and morbidity. Bull World Health Organ. 2010;88(1):31–8. 10.2471/BLT.08.062554 20428351 PMC2802437

[9] Khan KS, Wojdyla D, Say L, Gulmezoglu AM, Van Look PF. WHO analysis of causes of maternal death: a systematic review. Lancet. 2006;367(9516):1066–74. 10.1016/S0140-6736(06)68397-9 . 16581405

[10] Melin P, Efstratiou A. Group B streptococcal epidemiology and vaccine needs in developed countries. Vaccine. 2013;31 Suppl 4:D31–42. 10.1016/j.vaccine.2013.05.012 . 23973345

[11] Schrag SJ, Zywicki S, Farley MM, Reingold AL, Harrison LH, Lefkowitz LB, et al Group B streptococcal disease in the era of intrapartum antibiotic prophylaxis. N Engl J Med. 2000;342(1):15–20. 10.1056/NEJM200001063420103 . 10620644

[12] Regan JA, Klebanoff MA, Nugent RP, Eschenbach DA, Blackwelder WC, Lou Y, et al Colonization with group B streptococci in pregnancy and adverse outcome. VIP Study Group. Am J Obstet Gynecol. 1996;174(4):1354–60. . 8623869 10.1016/s0002-9378(96)70684-1

[13] Ohlsson A, Shah VS. Intrapartum antibiotics for known maternal Group B streptococcal colonization. Cochrane Database Syst Rev. 2014;6:CD007467 10.1002/14651858.CD007467.pub4 . 24915629

[14] Verani JR, McGee L, Schrag SJ, Division of Bacterial Diseases NCfI, Respiratory Diseases CfDC, Prevention. Prevention of perinatal group B streptococcal disease—revised guidelines from CDC, 2010. MMWR Recomm Rep. 2010;59(RR-10):1–36. . 21088663

[15] Johri AK, Paoletti LC, Glaser P, Dua M, Sharma PK, Grandi G, et al Group B Streptococcus: global incidence and vaccine development. Nat Rev Microbiol. 2006;4(12):932–42. 10.1038/nrmicro1552 17088932 PMC2742968

[16] Capan M, Mombo-Ngoma G, Akerey-Diop D, Basra A, Wurbel H, Lendamba W, et al Epidemiology and management of group B streptococcal colonization during pregnancy in Africa. Wien Klin Wochenschr. 2012;124 Suppl 3:14–6. 10.1007/s00508-012-0239-5 . 23064861

[17] Stoll BJ, Schuchat A. Maternal carriage of group B streptococci in developing countries. Pediatr Infect Dis J. 1998;17(6):499–503. . 9655542 10.1097/00006454-199806000-00013

[18] Gautam R, Borgdorff H, Jespers V, Francis SC, Verhelst R, Mwaura M, et al Correlates of the molecular vaginal microbiota composition of African women. BMC Infect Dis. 2015;15:86 10.1186/s12879-015-0831-1 25887567 PMC4343073

[19] Jespers V, Crucitti T, Menten J, Verhelst R, Mwaura M, Mandaliya K, et al Prevalence and correlates of bacterial vaginosis in different sub-populations of women in sub-Saharan Africa: a cross-sectional study. PLoS One. 2014;9(10):e109670 10.1371/journal.pone.0109670 25289640 PMC4188821

[20] Jespers V, van de Wijgert J, Cools P, Verhelst R, Verstraelen H, Delany-Moretlwe S, et al The significance of Lactobacillus crispatus and L. vaginalis for vaginal health and the negative effect of recent sex: a cross-sectional descriptive study across groups of African women. BMC Infect Dis. 2015;15:115 10.1186/s12879-015-0825-z 25879811 PMC4351943

[21] Kyongo JK, Crucitti T, Menten J, Hardy L, Cools P, Michiels J, et al Cross-Sectional Analysis of Selected Genital Tract Immunological Markers and Molecular Vaginal Microbiota in Sub-Saharan African Women, with Relevance to HIV Risk and Prevention. Clin Vaccine Immunol. 2015;22(5):526–38. 10.1128/CVI.00762-14 25761460 PMC4412937

[22] Nugent RP, Krohn MA, Hillier SL. Reliability of diagnosing bacterial vaginosis is improved by a standardized method of gram stain interpretation. J Clin Microbiol. 1991;29(2):297–301. 1706728 10.1128/jcm.29.2.297-301.1991 PMC269757

[23] Bergh K, Stoelhaug A, Loeseth K, Bevanger L. Detection of group B streptococci (GBS) in vaginal swabs using real-time PCR with TaqMan probe hybridization. Indian J Med Res. 2004;119 Suppl:221–3. . 15232199

[24] Chern EC, Siefring S, Paar J, Doolittle M, Haugland RA. Comparison of quantitative PCR assays for Escherichia coli targeting ribosomal RNA and single copy genes. Lett Appl Microbiol. 2011;52(3):298–306. 10.1111/j.1472-765X.2010.03001.x . 21204885

[25] Guiver M, Levi K, Oppenheim BA. Rapid identification of candida species by TaqMan PCR. J Clin Pathol. 2001;54(5):362–6. 11328834 10.1136/jcp.54.5.362 PMC1731427

[26] Rabe LK, Hillier SL. Optimization of media for detection of hydrogen peroxide production by Lactobacillus species. J Clin Microbiol. 2003;41(7):3260–4. 12843073 10.1128/JCM.41.7.3260-3264.2003 PMC165346

[27] Verhelst R, Verstraelen H, Claeys G, Verschraegen G, Delanghe J, Van Simaey L, et al Cloning of 16S rRNA genes amplified from normal and disturbed vaginal microflora suggests a strong association between Atopobium vaginae, Gardnerella vaginalis and bacterial vaginosis. BMC Microbiol. 2004;4:16 10.1186/1471-2180-4-16 15102329 PMC419343

[28] Yao K, Poulsen K, Maione D, Rinaudo CD, Baldassarri L, Telford JL, et al Capsular gene typing of Streptococcus agalactiae compared to serotyping by latex agglutination. J Clin Microbiol. 2013;51(2):503–7. 10.1128/JCM.02417-12 23196363 PMC3553911

[29] Poyart C, Tazi A, Reglier-Poupet H, Billoet A, Tavares N, Raymond J, et al Multiplex PCR assay for rapid and accurate capsular typing of group B streptococci. J Clin Microbiol. 2007;45(6):1985–8. 10.1128/JCM.00159-07 17376884 PMC1933079

[30] Imperi M, Pataracchia M, Alfarone G, Baldassarri L, Orefici G, Creti R. A multiplex PCR assay for the direct identification of the capsular type (Ia to IX) of Streptococcus agalactiae. J Microbiol Methods. 2010;80(2):212–4. 10.1016/j.mimet.2009.11.010 . 19958797

[31] Macaluso M, Lawson L, Akers R, Valappil T, Hammond K, Blackwell R, et al Prostate-specific antigen in vaginal fluid as a biologic marker of condom failure. Contraception. 1999;59(3):195–201. . 10382083 10.1016/s0010-7824(99)00013-x

[32] Babyak MA. What you see may not be what you get: a brief, nontechnical introduction to overfitting in regression-type models. Psychosom Med. 2004;66(3):411–21. . 15184705 10.1097/01.psy.0000127692.23278.a9

[33] Wakkee M, Hollestein LM, Nijsten T. Multivariable analysis. J Invest Dermatol. 2014;134(5):e20; quiz e. 10.1038/jid.2014.132 . 24732339

[34] Suara RO, Adegbola RA, Baker CJ, Secka O, Mulholland EK, Greenwood BM. Carriage of group B Streptococci in pregnant Gambian mothers and their infants. J Infect Dis. 1994;170(5):1316–9. . 7963736 10.1093/infdis/170.5.1316

[35] Gray KJ, Kafulafula G, Matemba M, Kamdolozi M, Membe G, French N. Group B Streptococcus and HIV infection in pregnant women, Malawi, 2008–2010. Emerg Infect Dis. 2011;17(10):1932–5. 10.3201/eid1710.102008 22000375 PMC3310663

[36] Madzivhandila M, Adrian PV, Cutland CL, Kuwanda L, Schrag SJ, Madhi SA. Serotype distribution and invasive potential of group B streptococcus isolates causing disease in infants and colonizing maternal-newborn dyads. PLoS One. 2011;6(3):e17861 10.1371/journal.pone.0017861 21445302 PMC3061872

[37] Kwatra G, Adrian PV, Shiri T, Buchmann EJ, Cutland CL, Madhi SA. Serotype-specific acquisition and loss of group B streptococcus recto-vaginal colonization in late pregnancy. PLoS One. 2014;9(6):e98778 10.1371/journal.pone.0098778 24979575 PMC4076185

[38] Ippolito DL, James WA, Tinnemore D, Huang RR, Dehart MJ, Williams J, et al Group B streptococcus serotype prevalence in reproductive-age women at a tertiary care military medical center relative to global serotype distribution. BMC Infect Dis. 2010;10:336 10.1186/1471-2334-10-336 21106080 PMC3004907

[39] Kim EJ, Oh KY, Kim MY, Seo YS, Shin JH, Song YR, et al Risk factors for group B streptococcus colonization among pregnant women in Korea. Epidemiol Health. 2011;33:e2011010 10.4178/epih/e2011010 22111030 PMC3221034

[40] Regan JA, Klebanoff MA, Nugent RP. The epidemiology of group B streptococcal colonization in pregnancy. Vaginal Infections and Prematurity Study Group. Obstet Gynecol. 1991;77(4):604–10. . 2002986

[41] Hastings MJ, Easmon CS, Neill J, Bloxham B, Rivers RP. Group B streptococcal colonisation and the outcome of pregnancy. J Infect. 1986;12(1):23–9. . 3514769 10.1016/s0163-4453(86)94775-4

[42] Valkenburg-van den Berg AW, Sprij AJ, Oostvogel PM, Mutsaers JA, Renes WB, Rosendaal FR, et al Prevalence of colonisation with group B Streptococci in pregnant women of a multi-ethnic population in The Netherlands. Eur J Obstet Gynecol Reprod Biol. 2006;124(2):178–83. 10.1016/j.ejogrb.2005.06.007 . 16026920

[43] Moyo SR, Mudzori J, Tswana SA, Maeland JA. Prevalence, capsular type distribution, anthropometric and obstetric factors of group B Streptococcus (Streptococcus agalactiae) colonization in pregnancy. Cent Afr J Med. 2000;46(5):115–20. . 11210331 10.4314/cajm.v46i5.8533

[44] Baker CJ, Goroff DK, Alpert S, Crockett VA, Zinner SH, Evrard JR, et al Vaginal colonization with group B streptococcus: a study in college women. J Infect Dis. 1977;135(3):392–7. . 321702 10.1093/infdis/135.3.392

[45] Baker CJ, Barrett FF, Yow MD. The influence of advancing gestation on group B streptococcal colonization in pregnant women. Am J Obstet Gynecol. 1975;122(7):820–3. . 1096618 10.1016/0002-9378(75)90721-8

[46] Hadavand S, Ghafoorimehr F, Rajabi L, Davati A, Zafarghandi N. Frequency of Group B Streptococcal Colonization in Pregnant Women Aged 35–37 Weeks in Clinical Centers of Shahed University, Tehran, Iran. Iran J Pathol. 2015;10(2):120–6. 26351472 PMC4539761

[47] Javanmanesh F, Eshraghi N. Prevalence of positive recto-vaginal culture for Group B streptococcus in pregnant women at 35–37 weeks of gestation. Med J Islam Repub Iran. 2013;27(1):7–11. 23483827 PMC3592944

[48] Gupta C, Briski LE. Comparison of two culture media and three sampling techniques for sensitive and rapid screening of vaginal colonization by group B streptococcus in pregnant women. J Clin Microbiol. 2004;42(9):3975–7. 10.1128/JCM.42.9.3975-3977.2004 15364978 PMC516316

[49] El Aila NA, Tency I, Claeys G, Saerens B, Cools P, Verstraelen H, et al Comparison of different sampling techniques and of different culture methods for detection of group B streptococcus carriage in pregnant women. BMC Infect Dis. 2010;10:285 10.1186/1471-2334-10-285 20920213 PMC2956727

[50] El Aila NA, Tency I, Claeys G, Verstraelen H, Deschaght P, Decat E, et al Comparison of culture with two different qPCR assays for detection of rectovaginal carriage of Streptococcus agalactiae (group B streptococci) in pregnant women. Res Microbiol. 2011;162(5):499–505. 10.1016/j.resmic.2011.04.001 . 21514378

[51] Davies HD, Miller MA, Faro S, Gregson D, Kehl SC, Jordan JA. Multicenter study of a rapid molecular-based assay for the diagnosis of group B Streptococcus colonization in pregnant women. Clin Infect Dis. 2004;39(8):1129–35. 10.1086/424518 . 15486835

[52] Convert M, Martinetti Lucchini G, Dolina M, Piffaretti JC. Comparison of LightCycler PCR and culture for detection of group B streptococci from vaginal swabs. Clin Microbiol Infect. 2005;11(12):1022–6. 10.1111/j.1469-0691.2005.01275.x . 16307558

[53] Rallu F, Barriga P, Scrivo C, Martel-Laferriere V, Laferriere C. Sensitivities of antigen detection and PCR assays greatly increased compared to that of the standard culture method for screening for group B streptococcus carriage in pregnant women. J Clin Microbiol. 2006;44(3):725–8. 10.1128/JCM.44.3.725-728.2006 16517846 PMC1393163

[54] Joachim A, Matee MI, Massawe FA, Lyamuya EF. Maternal and neonatal colonisation of group B streptococcus at Muhimbili National Hospital in Dar es Salaam, Tanzania: prevalence, risk factors and antimicrobial resistance. BMC Public Health. 2009;9:437 10.1186/1471-2458-9-437 19948075 PMC2791767

[55] Onile BA. Group B streptococcal carriage in Nigeria. Trans R Soc Trop Med Hyg. 1980;74(3):367–70. . 7001689 10.1016/0035-9203(80)90102-9

[56] Dawodu AH, Damole IO, Onile BA. Epidemiology of group B streptococcal carriage among pregnant women and their neonates: an African experience. Trop Geogr Med. 1983;35(2):145–50. . 6351383

[57] Nathoo KJ, Mason PR, Chimbira TH. Neonatal septicaemia in Harare Hospital: aetiology and risk factors. The Puerperal Sepsis Study Group. Cent Afr J Med. 1990;36(6):150–6. . 2261631

[58] David-Prince M, Ategbo S, De Souza AE, Eklu-Avlassu EK, Grunitzky-Bekele M, Schmidt-Ehry G, et al [Carriage of Streptococcus B in the mother and infant pair at birth. Apropos of 106 cases]. Bull Soc Pathol Exot. 1991;84(5 Pt 5):522–31. . 1819402

[59] Dzowela TK OO; Igbigbi A. Prevalence of group B Streptococcus colonization in antenatal women at the Queen Elizabeth Central Hospital Blantyre—a preliminary study. Malawi Medical Journal. 2005;17(3):97–9.

[60] de Steenwinkel FD, Tak HV, Muller AE, Nouwen JL, Oostvogel PM, Mocumbi SM. Low carriage rate of group B streptococcus in pregnant women in Maputo, Mozambique. Trop Med Int Health. 2008;13(3):427–9. 10.1111/j.1365-3156.2008.02018.x . 18397403

[61] Mavenyengwa RT, Afset JE, Schei B, Berg S, Caspersen T, Bergseng H, et al Group B Streptococcus colonization during pregnancy and maternal-fetal transmission in Zimbabwe. Acta Obstet Gynecol Scand. 2010;89(2):250–5. 10.3109/00016340903398029 . 19916889

[62] Mitima KT, Ntamako S, Birindwa AM, Mukanire N, Kivukuto JM, Tsongo K, et al Prevalence of colonization by Streptococcus agalactiae among pregnant women in Bukavu, Democratic Republic of the Congo. J Infect Dev Ctries. 2014;8(9):1195–200. 10.3855/jidc.5030 . 25212085

[63] Karou SD, Djigma F, Sagna T, Nadembega C, Zeba M, Kabre A, et al Antimicrobial resistance of abnormal vaginal discharges microorganisms in Ouagadougou, Burkina Faso. Asian Pac J Trop Biomed. 2012;2(4):294–7. 10.1016/S2221-1691(12)60025-2 23569916 PMC3609289

[64] Ekwempu CC, Lawande RV, Egler LJ. Microbial flora of the lower genital tract of women in labour in Zaria, Nigeria. J Clin Pathol. 1981;34(1):82–3. 7007447 10.1136/jcp.34.1.82 PMC1146417

[65] Schellenberg JJ, Links MG, Hill JE, Dumonceaux TJ, Kimani J, Jaoko W, et al Molecular definition of vaginal microbiota in East African commercial sex workers. Appl Environ Microbiol. 2011;77(12):4066–74. 10.1128/AEM.02943-10 21531840 PMC3131651

[66] Cutland CL, Schrag SJ, Zell ER, Kuwanda L, Buchmann E, Velaphi SC, et al Maternal HIV infection and vertical transmission of pathogenic bacteria. Pediatrics. 2012;130(3):e581–90. 10.1542/peds.2011-1548 . 22869824

[67] Sagna T, Djigma F, Zeba M, Bisseye C, Karou SD, Ouermi D, et al Human papillomaviruses prevalence and genital co-infections in HIV-seropositive women in Ouagadougou (Burkina Faso). Pak J Biol Sci. 2010;13(19):951–5. . 21313918 10.3923/pjbs.2010.951.955

[68] Razzak MS, Al-Charrakh AH, Al-Greitty BH. Relationship between lactobacilli and opportunistic bacterial pathogens associated with vaginitis. N Am J Med Sci. 2011;3(4):185–92. 10.4297/najms.2011.3185 22540089 PMC3336910

[69] Mobasheri M, Saeedi Varnamkhast N, Karimi A, Banaeiyan S. Prevalence study of genital tract infections in pregnant women referred to health centers in Iran. Turk J Med Sci. 2014;44(2):232–6. . 25536729 10.3906/sag-1208-33

[70] Obata-Yasuoka M, Ba-Thein W, Tsukamoto T, Yoshikawa H, Hayashi H. Vaginal Escherichia coli share common virulence factor profiles, serotypes and phylogeny with other extraintestinal E. coli. Microbiology. 2002;148(Pt 9):2745–52. . 12213921 10.1099/00221287-148-9-2745

[71] Kazi YF, Saleem S, Kazi N. Investigation of vaginal microbiota in sexually active women using hormonal contraceptives in Pakistan. BMC Urol. 2012;12:22 10.1186/1471-2490-12-22 22901000 PMC3492163

[72] Ocak S, Cetin M, Hakverdi S, Dolapcioglu K, Gungoren A, Hakverdi AU. Effects of intrauterine device and oral contraceptive on vaginal flora and epithelium. Saudi Med J. 2007;28(5):727–31. . 17457440

[73] Kaliterna V, Kucisec-Tepes N, Pejkovic L, Zavorovic S, Petrovic S, Barisic Z. An intrauterine device as a possible cause of change in the microbial flora of the female genital system. J Obstet Gynaecol Res. 2011;37(8):1035–40. 10.1111/j.1447-0756.2010.01480.x . 21481090

[74] Stokholm J, Schjorring S, Eskildsen CE, Pedersen L, Bischoff AL, Folsgaard N, et al Antibiotic use during pregnancy alters the commensal vaginal microbiota. Clin Microbiol Infect. 2014;20(7):629–35. 10.1111/1469-0691.12411 . 24118384

[75] Chase DJ, Schenkel BP, Fahr AM, Eigner U, Tampon Study G. A prospective, randomized, double-blind study of vaginal microflora and epithelium in women using a tampon with an apertured film cover compared with those in women using a commercial tampon with a cover of nonwoven fleece. J Clin Microbiol. 2007;45(4):1219–24. 10.1128/JCM.02156-06 17287327 PMC1865837

[76] Iavazzo C, Vogiatzi C, Falagas ME. A retrospective analysis of isolates from patients with vaginitis in a private Greek obstetric/gynecological hospital (2003–2006). Med Sci Monit. 2008;14(4):CR228–31. . 18376352

[77] Tameliene R, Barcaite E, Stoniene D, Buinauskiene J, Markuniene E, Kudreviciene A, et al Escherichia coli colonization in neonates: prevalence, perinatal transmission, antimicrobial susceptibility, and risk factors. Medicina (Kaunas). 2012;48(2):71–6. . 22491384

[78] Bayo M, Berlanga M, Agut M. Vaginal microbiota in healthy pregnant women and prenatal screening of group B streptococci (GBS). Int Microbiol. 2002;5(2):87–90. . 12180785 10.1007/s10123-002-0064-1

[79] Guiral E, Bosch J, Vila J, Soto SM. Prevalence of Escherichia coli among samples collected from the genital tract in pregnant and nonpregnant women: relationship with virulence. FEMS Microbiol Lett. 2011;314(2):170–3. 10.1111/j.1574-6968.2010.02160.x . 21133987

[80] Nikolaitchouk N, Andersch B, Falsen E, Strombeck L, Mattsby-Baltzer I. The lower genital tract microbiota in relation to cytokine-, SLPI- and endotoxin levels: application of checkerboard DNA-DNA hybridization (CDH). APMIS. 2008;116(4):263–77. 10.1111/j.1600-0463.2008.00808.x . 18397461

[81] Percival-Smith R, Bartlett KH, Chow AW. Vaginal colonization of Escherichia coli and its relation to contraceptive methods. Contraception. 1983;27(5):497–504. . 6349927 10.1016/0010-7824(83)90046-x

[82] Eschenbach DA, Patton DL, Hooton TM, Meier AS, Stapleton A, Aura J, et al Effects of vaginal intercourse with and without a condom on vaginal flora and vaginal epithelium. J Infect Dis. 2001;183(6):913–8. 10.1086/319251 . 11237808

[83] Hyman RW, Fukushima M, Diamond L, Kumm J, Giudice LC, Davis RW. Microbes on the human vaginal epithelium. Proc Natl Acad Sci U S A. 2005;102(22):7952–7. 10.1073/pnas.0503236102 15911771 PMC1142396

[84] Ghartey JP, Carpenter C, Gialanella P, Rising C, McAndrew TC, Mhatre M, et al Association of bactericidal activity of genital tract secretions with Escherichia coli colonization in pregnancy. Am J Obstet Gynecol. 2012;207(4):297 e1-8. 10.1016/j.ajog.2012.07.025 22867687 PMC3462306

[85] Anderson BL, Mendez-Figueroa H, Dahlke JD, Raker C, Hillier SL, Cu-Uvin S. Pregnancy-induced changes in immune protection of the genital tract: defining normal. Am J Obstet Gynecol. 2013;208(4):321 e1-9. 10.1016/j.ajog.2013.01.014 23313311 PMC3610848

[86] Villar HE, Aubert V, Baserni MN, Jugo MB. Maternal carriage of extended-spectrum beta-lactamase-producing Escherichia coli isolates in Argentina. J Chemother. 2013;25(6):324–7. 10.1179/1973947813Y.0000000081 . 24091027

[87] Lobos O, Padilla C. Phenotypic characterization and genomic DNA polymorphisms of Escherichia coli strains isolated as the sole micro-organism from vaginal infections. Microbiology. 2009;155(Pt 3):825–30. 10.1099/mic.0.021733-0 . 19246753

[88] Cotch MF, Hillier SL, Gibbs RS, Eschenbach DA. Epidemiology and outcomes associated with moderate to heavy Candida colonization during pregnancy. Vaginal Infections and Prematurity Study Group. Am J Obstet Gynecol. 1998;178(2):374–80. . 9500502 10.1016/s0002-9378(98)80028-8

[89] Beigi RH, Meyn LA, Moore DM, Krohn MA, Hillier SL. Vaginal yeast colonization in nonpregnant women: a longitudinal study. Obstet Gynecol. 2004;104(5 Pt 1):926–30. 10.1097/01.AOG.0000140687.51048.73 . 15516380

[90] Low N, Chersich MF, Schmidlin K, Egger M, Francis SC, van de Wijgert JH, et al Intravaginal practices, bacterial vaginosis, and HIV infection in women: individual participant data meta-analysis. PLoS Med. 2011;8(2):e1000416 10.1371/journal.pmed.1000416 21358808 PMC3039685

[91] van De Wijgert JH, Mason PR, Gwanzura L, Mbizvo MT, Chirenje ZM, Iliff V, et al Intravaginal practices, vaginal flora disturbances, and acquisition of sexually transmitted diseases in Zimbabwean women. J Infect Dis. 2000;181(2):587–94. 10.1086/315227 . 10669342

[92] Foxman B, de Azevedo CL, Buxton M, DeBusscher J, Pillai P, De Carvalho NS, et al Acquisition and transmission of group B Streptococcus during pregnancy. J Infect Dis. 2008;198(9):1375–8. 10.1086/592221 . 18774883

[93] Foxman B, Gillespie BW, Manning SD, Marrs CF. Risk factors for group B streptococcal colonization: potential for different transmission systems by capsular type. Ann Epidemiol. 2007;17(11):854–62. 10.1016/j.annepidem.2007.05.014 17689259 PMC2099698

[94] Honig E, Mouton JW, van der Meijden WI. The epidemiology of vaginal colonisation with group B streptococci in a sexually transmitted disease clinic. Eur J Obstet Gynecol Reprod Biol. 2002;105(2):177–80. . 12381483 10.1016/s0301-2115(02)00162-8

[95] Ravel J, Brotman RM, Gajer P, Ma B, Nandy M, Fadrosh DW, et al Daily temporal dynamics of vaginal microbiota before, during and after episodes of bacterial vaginosis. Microbiome. 2013;1(1):29 10.1186/2049-2618-1-29 24451163 PMC3968321

[96] Foxman B, Gillespie B, Manning SD, Howard LJ, Tallman P, Zhang L, et al Incidence and duration of group B Streptococcus by serotype among male and female college students living in a single dormitory. Am J Epidemiol. 2006;163(6):544–51. 10.1093/aje/kwj075 . 16421237

[97] Lee V, Tobin JM, Foley E. Relationship of cervical ectopy to chlamydia infection in young women. J Fam Plann Reprod Health Care. 2006;32(2):104–6. 10.1783/147118906776276440 . 16824301

[98] Rocha-Zavaleta L, Yescas G, Cruz RM, Cruz-Talonia F. Human papillomavirus infection and cervical ectopy. Int J Gynaecol Obstet. 2004;85(3):259–66. 10.1016/j.ijgo.2003.10.002 . 15145262

[99] Venkatesh KK, Cu-Uvin S. Assessing the relationship between cervical ectopy and HIV susceptibility: implications for HIV prevention in women. Am J Reprod Immunol. 2013;69 Suppl 1:68–73. 10.1111/aji.12029 . 23057756

[100] De Luca Brunori I, Facchini V, Filippeschi M, Battini L, Giusti G, Romani L, et al Cell-mediated immunity in the course of cervical ectropion. Clin Exp Obstet Gynecol. 1994;21(2):105–7. . 7915218

[101] Veldhuijzen NJ, Ingabire C, Luchters S, Bosire W, Braunstein S, Chersich M, et al Anal intercourse among female sex workers in East Africa is associated with other high-risk behaviours for HIV. Sex Health. 2011;8(2):251–4. 10.1071/SH10047 . 21592442

[102] Ghanem KG, Hutton HE, Zenilman JM, Zimba R, Erbelding EJ. Audio computer assisted self interview and face to face interview modes in assessing response bias among STD clinic patients. Sex Transm Infect. 2005;81(5):421–5. 10.1136/sti.2004.013193 16199744 PMC1745029

[103] Schwandt M, Morris C, Ferguson A, Ngugi E, Moses S. Anal and dry sex in commercial sex work, and relation to risk for sexually transmitted infections and HIV in Meru, Kenya. Sex Transm Infect. 2006;82(5):392–6. 10.1136/sti.2006.019794 16790563 PMC2563859

[104] Stoll BJ, Hansen NI, Sanchez PJ, Faix RG, Poindexter BB, Van Meurs KP, et al Early onset neonatal sepsis: the burden of group B Streptococcal and E. coli disease continues. Pediatrics. 2011;127(5):817–26. 10.1542/peds.2010-2217 21518717 PMC3081183

[105] Joubrel C, Tazi A, Six A, Dmytruk N, Touak G, Bidet P, et al Group B streptococcus neonatal invasive infections, France 2007–2012. Clin Microbiol Infect. 2015;21(10):910–6. 10.1016/j.cmi.2015.05.039 . 26055414

[106] Afshar B, Broughton K, Creti R, Decheva A, Hufnagel M, Kriz P, et al International external quality assurance for laboratory identification and typing of Streptococcus agalactiae (Group B streptococci). J Clin Microbiol. 2011;49(4):1475–82. 10.1128/JCM.02365-10 21325542 PMC3122801

[107] Brigtsen AK, Dedi L, Melby KK, Holberg-Petersen M, Radtke A, Lyng RV, et al Comparison of PCR and serotyping of Group B Streptococcus in pregnant women: the Oslo GBS-study. J Microbiol Methods. 2015;108:31–5. 10.1016/j.mimet.2014.11.001 . 25447890

[108] Hillier SL, Krohn MA, Nugent RP, Gibbs RS. Characteristics of three vaginal flora patterns assessed by gram stain among pregnant women. Vaginal Infections and Prematurity Study Group. Am J Obstet Gynecol. 1992;166(3):938–44. . 1372474 10.1016/0002-9378(92)91368-k

[109] Rocchetti TT, Marconi C, Rall VL, Borges VT, Corrente JE, da Silva MG. Group B streptococci colonization in pregnant women: risk factors and evaluation of the vaginal flora. Arch Gynecol Obstet. 2011;283(4):717–21. 10.1007/s00404-010-1439-8 . 20349243

